# Structural Insights into Pixatimod (PG545) Inhibition of Heparanase, a Key Enzyme in Cancer and Viral Infections

**DOI:** 10.1002/chem.202104222

**Published:** 2022-01-31

**Authors:** Mohit Chhabra, Jennifer C. Wilson, Liang Wu, Gideon J. Davies, Neha S. Gandhi, Vito Ferro

**Affiliations:** ^1^ School of Chemistry & Molecular Biosciences The University of Queensland Brisbane Queensland 4072 Australia; ^2^ Australian Infectious Diseases Research Centre The University of Queensland Brisbane Queensland 4072 Australia; ^3^ School of Pharmacy and Medical Science Griffith University Gold Coast Campus Queensland Australia; ^4^ The Rosalind Franklin Institute Harwell Campus Didcot OX11 0FA UK; ^5^ Department of Chemistry University of York Heslington York YO10 5DD UK; ^6^ Centre for Genomics and Personalised Health School of Chemistry and Physics Queensland University of Technology 2 George St Brisbane QLD 4000 Australia

**Keywords:** conformational analysis, heparan sulfate, heparanase, molecular dynamics simulations, pixatimod (PG545)

## Abstract

Pixatimod (PG545), a heparan sulfate (HS) mimetic and anticancer agent currently in clinical trials, is a potent inhibitor of heparanase. Heparanase is an *endo*‐β‐glucuronidase that degrades HS in the extracellular matrix and basement membranes and is implicated in numerous pathological processes such as cancer and viral infections, including SARS−CoV‐2. To understand how PG545 interacts with heparanase, we firstly carried out a conformational analysis through a combination of NMR experiments and molecular modelling which showed that the reducing end β‐D‐glucose residue of PG545 adopts a distorted conformation. This was followed by docking and molecular dynamics simulations to study the interactions of PG545 with heparanase, revealing that PG545 is able to block the active site by binding in different conformations, with the cholestanol side‐chain making important hydrophobic interactions. While PG545 blocks its natural substrate HS from binding to the active site, small synthetic heparanase substrates are only partially excluded, and thus pentasaccharide or larger substrates are preferred for assaying this class of inhibitor. This study provides new insights for the design of next‐generation heparanase inhibitors and substrates.

## Graphical abstract

**Pixatimod (PG545)**, a drug candidate, is a potent inhibitor of heparanase. Conformational analysis by NMR spectroscopy and molecular dynamics simulations revealed a flexible reducing end residue in a skew boat‐like conformation. Docking and molecular dynamics simulations of PG545 with heparanase show that PG545 can block the active site by binding in different conformations, with the cholestanol sidechain making important hydrophobic interactions.

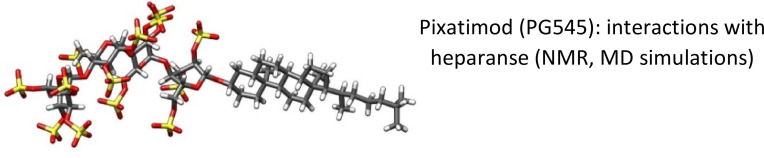

## Introduction

**Figure 1:**
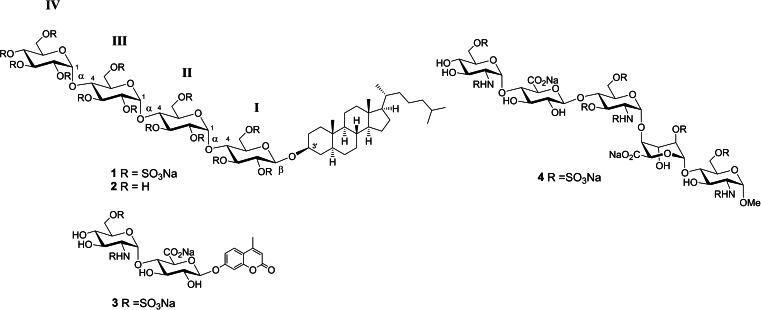
Structures of pixatimod (PG545) **1**, showing labelling of D‐glucose residues (I–IV) from the reducing end, and other compounds considered in this study (**2**–**4**).

Heparanase is an endo‐β‐glucuronidase responsible for degradation of heparan sulfate (HS),^[1]^ a highly sulfated polysaccharide found on the mammalian cell surface and extracellular matrix (ECM). Degradation of HS by heparanase has been implicated in metastasis, tumor growth, angiogenesis,^[2]^ and viral pathogenesis,^[3]^ as well as numerous other pathologies^[4]^ where inflammation plays a role. Heparanase overexpression has been observed in all human tumours and is correlated with the angiogenic and metastatic activity of tumour cells.^[5]^ Heparanase is now a well‐established target for anticancer drug development.^[6]^ Several heparanase inhibitors have progressed to clinical trials, including pixatimod (PG545) **1** (Figure 1) which is currently in Phase 2 clinical trials in combination with nivolumab (ClinicalTrials.gov Identifier: NCT05061017).^[7]^ PG545 is an HS mimetic that exhibits multiple therapeutic activities from immunomodulatory to antiangiogenic activity through interaction with numerous angiogenic growth factors (for example, VEGF and FGF) and potent inhibition of heparanase. PG545 has also recently shown potent antiviral activity against a number of viruses,^[8]^ including SARS−CoV‐2,^[9]^ with its antiviral effects attributed, in part, to inhibition of heparanase. Studying the interactions of PG545 with heparanase is thus important to understand how this clinical candidate exerts its therapeutic effects. The outcomes could guide design of next‐generation heparanase inhibitors.

X‐ray crystal structures of human heparanase in the apo form (pdb code 5E8M) and in complex with several HS oligosaccharides have been determined.^[10]^ These X‐ray structures reveal how an endo‐acting binding cleft is exposed by proteolytic activation of latent proHPSE, and also confirm the presence of the catalytic residues Glu225 (acid/base) and Glu343 (nucleophile) in the enzyme binding cleft. Molecular docking and molecular dynamics (MD) simulations play an important role in drug discovery, and such studies can also be helpful for studying heparanase inhibitors. Based on the above crystal structures, several heparanase inhibitors have been modelled with heparanase and their interactions with heparin‐binding domains (HBDs) and the catalytic site were explored.^[11]^ These studies include the modified heparin polysaccharide roneparstat,^[11a]^ a synthetic HS glycopeptide,^[11d]^ sulfated glycopolymers^[11b]^ and small molecule inhibitors.^[11c]^ Active heparanase possesses hydrophobic pockets around the catalytic site, which potentially makes it a good target for amphiphilic molecules. PG545 contains lipophilic groups that can make van der Waals interactions with these hydrophobic residues.[^6c^, ^12^] Unfortunately, attempts to soak solutions of PG545 into heparanase crystals to obtain a co‐crystal complex were not successful and no electron density was observed for PG545 in the X‐ray crystal structure. Herein we describe the conformational analysis of PG545 as determined through NMR experiments in combination with molecular modelling. This is followed by docking and MD simulations to study the interactions of PG545 with heparanase, including simulations in the presence of a fluorogenic disaccharide substrate. These studies reveal new insights into how PG545 engages with its target and provide important information about the suitability of different types of heparanase assays for this class of inhibitor.

## Results and Discussion

### NMR analysis of PG545

**Figure 2:**
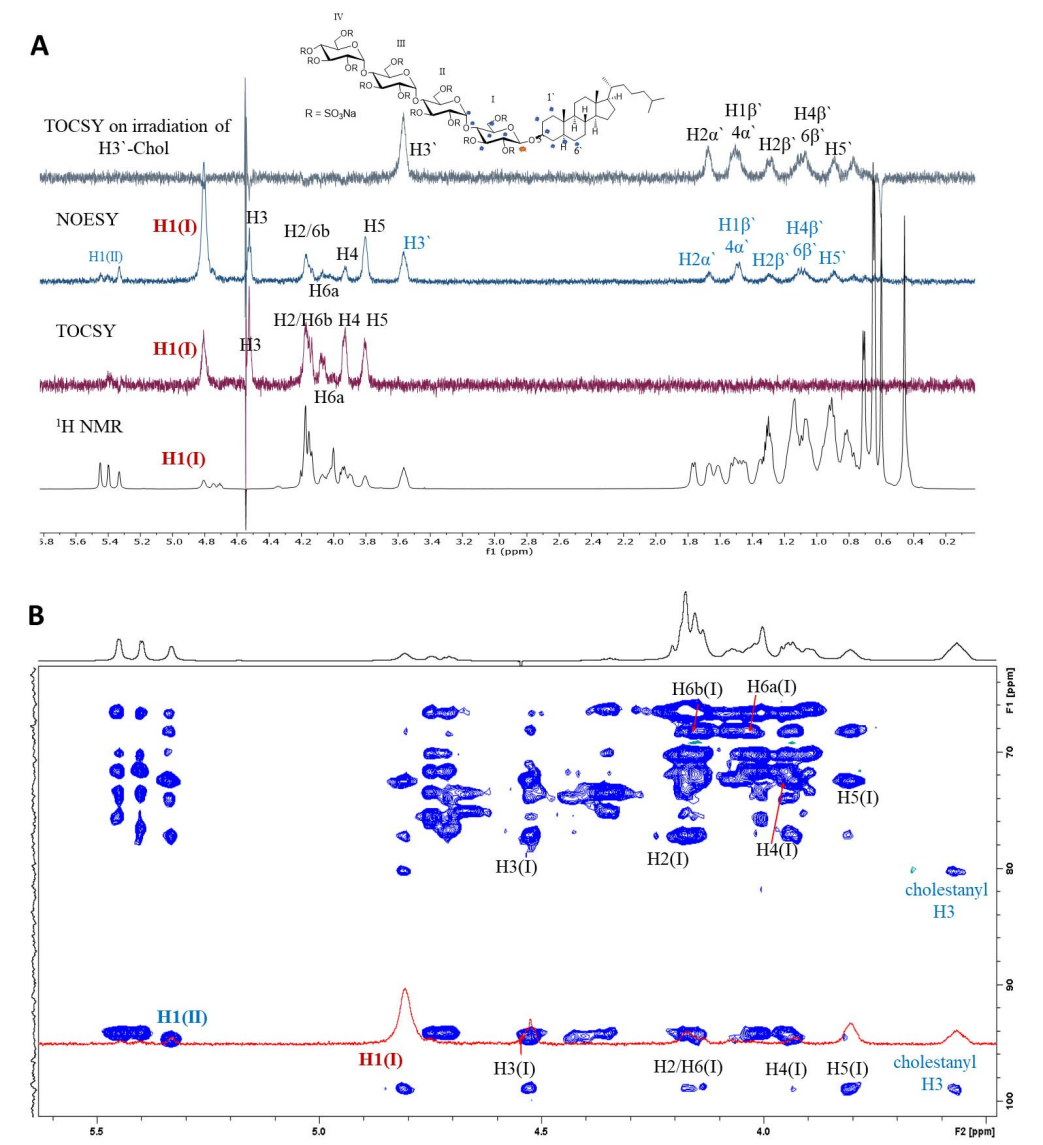
A) Overlaid ^1^H, 1D‐TOCSY and 1D‐NOESY spectra of PG545 with selective irradiation of H1 of sugar residue I, and 1D‐TOCSY following irradiation of H3’ of cholestanol (top). Black letters – intra‐residue correlations, blue letters – inter‐residue correlations in 1D‐NOESY. All NOE's are marked on the PG545 structure with blue * while the irradiated proton is marked with red *. B) 2D ^1^H‐^13^C HSQC‐TOCSY spectrum (blue) (600 MHz, 298 K) overlaid with 1D NOE spectrum on selective irradiation of H1 of residue I (at 4.81 ppm).

The ^1^H and ^13^C NMR chemical shifts for PG545 have been fully assigned previously.^[13]^ Herein PG545 was analysed for its intra‐ and inter‐residue connectivity through 1D and 2D NMR experiments and MD simulations. PG545 is a highly sulfated tetrasaccharide glycoside comprising α‐(1→4)‐glucose residues β‐linked to cholestanol (Figure 1). The ^1^H NMR signals for the anomeric protons of PG545 from residues II (5.34 ppm), III (5.40 ppm) and IV (5.45 ppm) show coupling constants *J*_1,2_ of 3.69, 3.73 and 3.37 Hz, respectively, confirming their α‐anomeric linkage. The signal for the β‐anomeric proton of residue I is located upfield as expected (4.81 ppm) but with an unusual coupling constant of 4.97 Hz (see below). Intra‐ and inter‐residue H−H interactions were obtained by selectively irradiating the anomeric proton of each sugar residue using one‐dimensional selective versions of the NOESY and TOCSY sequences. H1 of each sugar residue shows intra‐residual NOE's with H2−H6, and some inter‐residual NOE's to adjacent residues, which can be seen in overlaid spectra (Figure 2 for residue I, and Figure S1A–C, Supporting Information, for residues II–IV). Shown at the top of each of these figures are the ^1^H NMR spectra between 0–5.8 ppm overlaid with the selective 1D versions of the TOCSY and NOESY spectra, plus the 1D‐TOCSY spectrum from irradiation of a proton on an adjacent residue. The bottom of each figure shows the ^1^H−^13^C HSQC‐TOCSY spectrum used to aid assignment.

Selective irradiation of H1 of residue I shows strong intra‐residual NOEs with H3 and H5, medium strength NOEs for H2, H4 and H6b, and a weak NOE for 6a (Figure 2A). H1 also shows medium to weak inter‐residual NOE interactions with H1 of the adjacent residue II (∼5.4 ppm), and H3′(∼3.6 ppm), H1′β, H2′α/β, H4′α/β, H5′ and H6′β of cholestanol. The 1D selective TOCSY spectrum of residue I confirms intra‐residual connectivity from H1 of I to H2, H3, H4, H5 and H6. Residues II, III and IV were examined in a similar manner (see Figure S1A–C), confirming the expected intra‐ and inter‐residual connectivity. The H3′ signal of the cholestanol moiety at 3.6 ppm was resolved from all other sugar and steroid signals. Selective irradiation of the H3′ residue of cholestanol and application of a NOESY sequence gave the 1D NOE spectrum which was overlaid with the 2D NOESY spectrum (Figure S2). This provided a simple way to assess the intra‐ and inter‐residue connectivity of the cholestanol moiety with the sugar residues. H3′ of cholestanol shows extensive NOE interactions with residue I with a strong NOE to H1(I), and medium to weak NOEs with H2(I), H3(I), H4(I), H5(I), H6a/b(I), and with multiple cholestanol signals (for example, 1′α/β, 2′α/β, 4′α/β, 5′). Taken together there are many inter‐residue NOE's identified in the spectra shown in (Figures 2 and S1, S2) between the sugar residues I, II, III, IV, the sugar residues and cholestanol and within the cholestanol moiety that reflect the conformational preferences of PG545.

**Figure 3:**
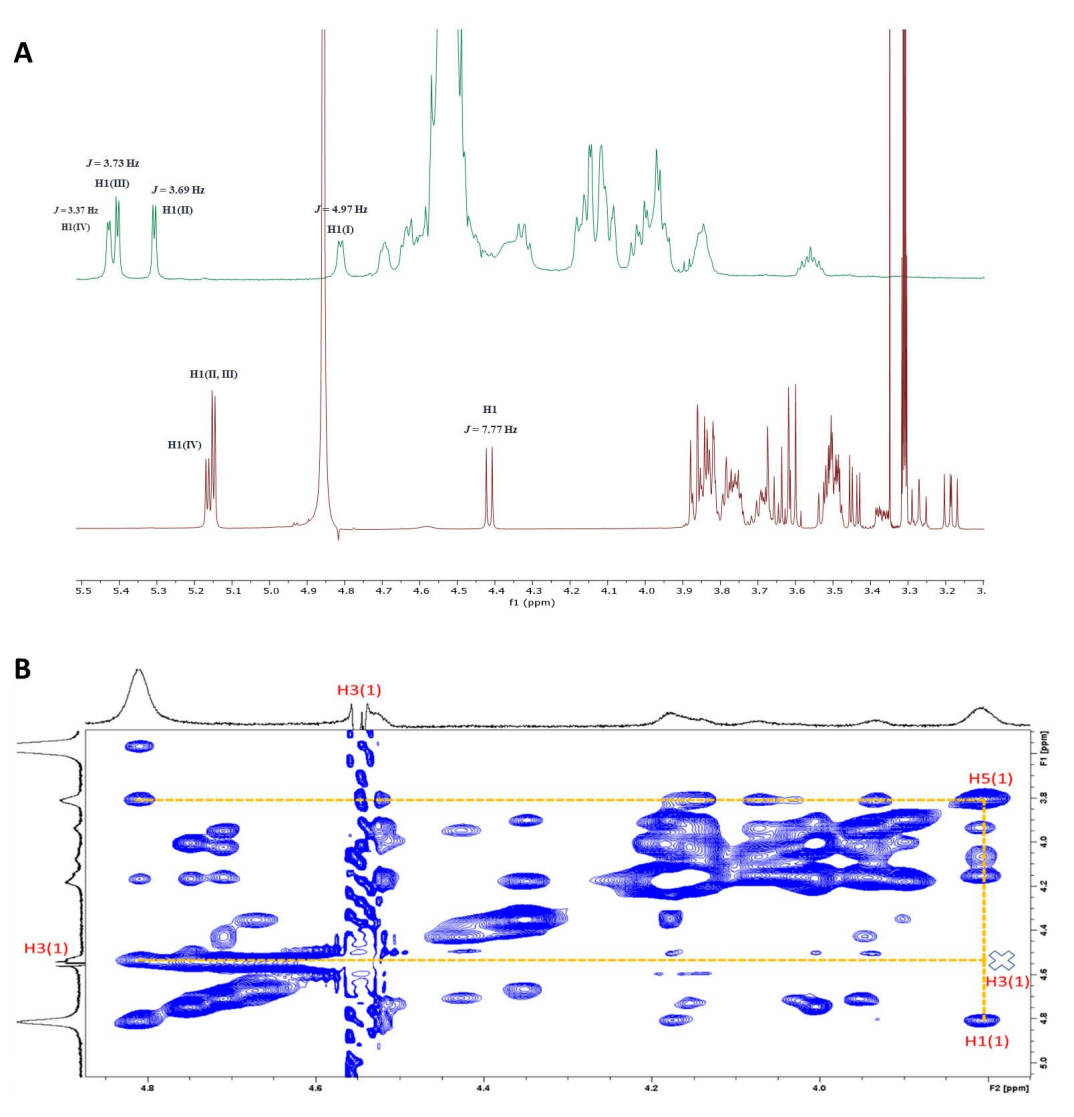
A) Stacked ^1^H NMR spectra of PG545 (**1**) (top‐green) and its non‐sulfated precursor (**2**) (bottom‐red); B) 2D NOESY spectrum of PG545.

Furthermore, intra‐ and inter‐residue H−H distances were calculated on the basis of the NOEs measured in the spectra and then the NMR data and MD simulations (see below) were used to investigate the conformation of PG545. The measured NOEs were classified as strong, medium, and weak (Table 1), and the H1−H2 (2.5 Å) distance of internal glucose residues was used as a reference distance. The H−H distances measured from the NMR data and the MD simulations were in good agreement, and together with the *J*_1,2_ values (3.37–3.73 Hz), are consistent with a ^4^ *C*_1_ conformation for internal residues. Unfortunately, the determination of other ^3^ *J*_HH_ coupling constants to support the conformation of these residues was not possible due to signal overlap and broadening. Importantly, the NMR spectroscopic data also support a distorted conformation for residue I of PG545, in accordance with the known conformational preferences of fully sulfated β‐D‐glucopyranosides (but not their corresponding non‐sulfated or α‐linked congeners).^[14]^ The signal for H1 appears as a doublet with *J*_1,2_=4.97 Hz which is similar to that reported for fully sulfated methyl β‐D‐glucoside (*J*_1,2_=5.0 Hz)^[14d]^ which displays a skew boat‐like conformation intermediate between ^3,O^ *B* and ^3^ *S*_1_. In comparison, the corresponding doublet for non‐sulfated PG545 (**2**)^[15]^ shows *J*_1,2_=7.77 Hz (Figure 3A) which is in the typical range for a ^4^ *C*_1_ chair conformation. Furthermore, strong H5−H1 and negligible H5−H3 NOEs (Figure 3B) confirm a distorted ring conformation (the H5−H3 NOE should be strong if the sugar was in a conventional ^4^ *C*_1_ conformation). This is further supported by the presence of a moderate NOE between H1−H2, which would also be negligible for a ^4^ *C*_1_ chair conformation.

**Table 1:** Intra‐ and Inter‐residue NOE data obtained for PG545. Distance constraints used for classification: strong 1.8–2.8 Å, medium 2.9–3.3 Å and weak 3.4–6.0 Å. The effective H−H distance rNOE was calculated from the peak integral *I* using the equation *r*_NOE_ *=r*_ref_ (*I*_ref_/*I*)^1/6^. * Virtual NOE distance, multiple H−H pairs contribute.

Atom pair	Experimental distance [Å]	MD simulation [Å] ± std. dev	Classification
H1(I)−H2(I)	2.5 (ref.)	2.6±0.15	Strong
H1(I)−H3(I)	4.0	4.2±0.11	Weak
H1(I)−H4(I)	4.5	4.8±0.20	Weak
H1(I)−H5(I)	3.5	3.7±0.60	Weak
H1(I)−H6(I)	4.1	4.3±0.35	Composite*
H1(I)‐ H3(chol)	3.2	2.9±0.35	Medium
H1(II)−H1(I)	6.0	6.0±0.30	Weak
H1(II)−H3(I)	3.1	3.1±0.60	Medium
H1(II)−H4(I)	2.7	2.4±0.23	Strong
H1(II)−H5(I)	3.8	3.9±0.54	Weak
H1(II)−H6a(I)	5.1	5.1±0.46	Composite*
H1(III)−H3(II)	3.1	3.7±0.65	Medium
H1(III)−H4(II)	3.0	2.7±0.33	Medium
H1(IV)−H3(III)	3.1	3.6±0.35	Medium
H1(IV)−H4(III)	2.9	2.7±0.32	Strong
H3(chol)−H2(chol)	2.3	2.3±0.09	Strong
H3(chol)−H1(I)	3.2	2.7±0.35	Medium
H3(chol)−H2(I)	3.7	3.9±0.89	Weak
H3(chol)−H4(I)	5.1	5.7±0.47	Composite*
H3(chol)−H5(I)	4.2	4.7±0.72	Composite*
H3(chol)−H6a(I)	3.8	4.2±1.15	Weak
H1(II)−H3(II)	3.8	3.8±0.11	Weak
H1(II)−H5(II)	3.8	3.7±0.10	Weak
H1(II)−H6(a/b) (II)	4.2	4.7±0.33	Composite*
H1(III)−H3(III)	3.7	3.8±0.11	Weak
H1(III)−H4(III)	4.3	4.1±0.16	Composite*

### MD simulations of PG545

**Figure 4:**
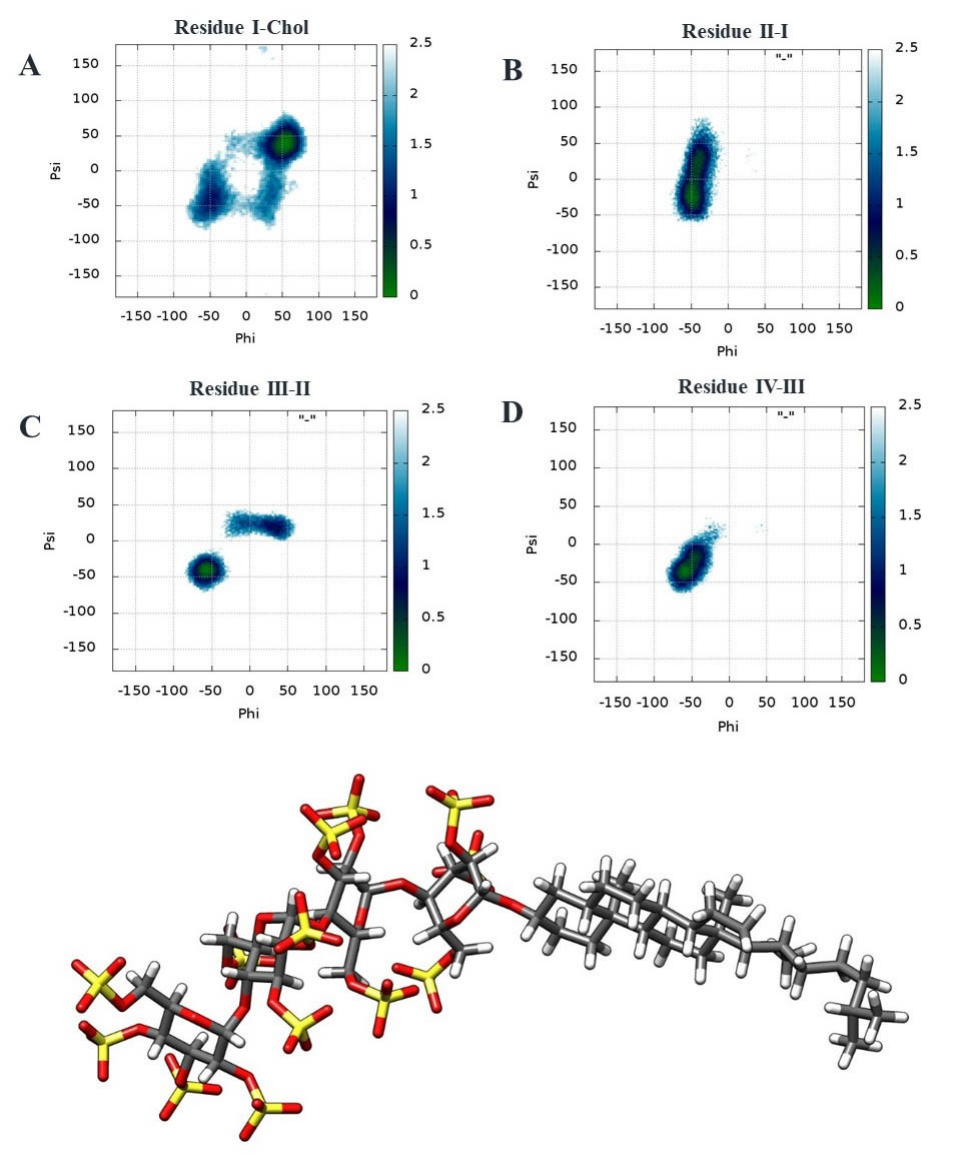
Top: Free energy surfaces (kcal/mol) of PG545 for the *ϕ*/*ψ* glycosidic angles of A) β‐D‐Glu(I)‐(1→3)‐Chol, B) α‐D‐Glu(II)‐(1→4)‐D‐Glu(I), C) α‐D‐Glu(III)‐(1→4)‐D‐Glu(II) and D) α‐D‐Glu(IV)‐(1→4)‐D‐Glu(III). Colour maps indicate the *ϕ*/*ψ* population with increasing trend from blue to green with green being the lowest energy state. Bottom: A representative minimum energy conformation of PG545 derived from MD simulations. Residue I is in a skew boat‐like conformation and residues II–IV are in ^4^ *C*_1_ conformations.

The glycosidic torsions of PG545 were investigated from MD simulations (400 ns production runs) (see Figure S3, Table S1). Two‐dimensional (2D) free energy surfaces of *ϕ*/*ψ* glycosidic torsions were constructed for the glycosidic linkages of α‐D‐Glu(IV)‐(1→4)‐D‐Glu(III), α‐D‐Glu(III)‐(1→4)‐D‐Glu(II), α‐D‐Glu(II)‐(1→4)‐D‐Glu(I) and β‐D‐Glu(I)‐(1→3′)‐Chol (Figure 4), and were analysed for the flexibility of glycosidic linkage across the residues. The free energy surfaces of PG545 in Figure 4 show a single and narrow free energy minimum for the disaccharides α‐D‐Glu(IV)‐(1→4)‐D‐Glu(III) (*ϕ*=−72.6, *ψ*=−31.8), and α‐D‐Glu(III)‐(1→4)‐D‐Glu(II) (*ϕ*=−73.7, *ψ*=−51.3). The glycosidic linkage for α‐D‐Glu(II)‐(1→4)‐D‐Glu(I) displays a significant degree of flexibility, associated with the presence of the local minimum of free energy characterized by the free energy levels below 0.5 kcal/mol. The minimum is located at *ϕ*=−46.7, *ψ*=19.7. Similarly, the minimum for β‐D‐Glu(I)‐(1→3)‐Chol is located at *ϕ*=27.3, *ψ*=50.6. Examples of the preferred conformations of PG545 obtained from the MD simulations with interglycosidic distances matching the corresponding restraints in Table 1 are depicted in Figures 4 (bottom) and S4.

A previous study^[16]^ has reported that neighboring glycosidic linkages are affected by the conformation of the sugar rings only to a minor extent. The free energy surface didn't provide insight into the ring conformation. Therefore, we analysed the ring puckering of the sugars by obtaining the average azimuthal angle *θ* and monitoring simulation time versus *θ* (Figure S5). Residue I is in equilibrium between two conformations as indicated by azimuthal angle *θ*=180° and 90°. We visualised and monitored the meridian angle *ϕ* when *θ*=90° (Figures 4 and S5). The results show a skew boat‐like conformation intermediate between ^3,O^ *B*, ^O^ *S*_2_ and ^3^ *S*_1_ for residue I. The data for residues II and III suggest the rings are in a ^4^ *C*_1_ conformation. The standard deviation of the azimuthal angle *θ* and the plot for residue IV suggest the ring flips between the ^4^ *C*_1_ and ^1^ *C*_4_ conformations as characterized by *θ* values of around 0° and 180°, respectively. The amplitude *Q* which is the magnitude of puckering did not fluctuate much (∼0.5) for all four rings.

### Inhibition of heparanase by PG545

The inhibition of heparanase by PG545 has been determined previously via the fondaparinux assay^[17]^ with *K*_i_=4.44–6 nM,[^13^, ^18^] or via an ultrafiltration assay^[19]^ which uses radiolabelled HS (IC_50_=40 nM).^[18]^ Using the fondaparinux assay, it was shown that PG545 inhibits heparanase with parabolic competitive kinetics,^[18]^ indicating that PG545 may bind to two sites on the enzyme, suggested to be the hydrophobic regions adjacent to the active site, with each interaction sufficient to completely inhibit catalysis. Recently, we synthesized a fluorogenic, 4‐methylumbelliferyl HS disaccharide (**3**) as a heparanase substrate^[20a]^ and showed that, while turnover was slow, it was still suitable for assaying heparanase activity and inhibitor screening. We thus utilized this new assay to test the kinetics of heparanase inhibition by PG545 and obtained a *K*_i_ of ∼12.9 μM, several orders of magnitude less potent than that obtained from the fondaparinux assay (Figure S6A). Given PG545 is typically considered a tight heparanase binder, we also modelled its inhibition of heparanase‐**3** processing using the ‘Morrison’ quadratic method,^[21]^ which can be used in cases where *K*_i_ is comparable to the enzyme concentration in the reaction. We calculated a very similar inhibition constant (*K_i_* =20.1 μM) using this method, confirming the poor performance of PG545 against fluorogenic substrate **3** (Figure S6B). These results suggest that PG545 binding to heparanase readily blocks access to the active site for large substrates (for example, HS or the pentasaccharide fondaparinux **4**), but is less effective against smaller substrates such as **3**.^[20]^ Heparanase assays that utilize large substrates^[22]^ are thus preferred for determining the inhibition kinetics of this class of inhibitor.

### Molecular modelling of PG545−heparanase interactions

The unsuccessful co‐crystallisation experiments with heparanase, coupled with the previously reported parabolic inhibition kinetics and the anomalous inhibition when assayed using disaccharide substrate **3**, prompted the investigation of the interactions of PG545 with heparanase via docking and MD simulations. We chose to use the crystal structure of heparanase in complex with a HS tetrasaccharide (PDB ID: 5E9C)^[10]^ as a template. Other crystal structures such 5E8M^[10]^ or 6ZDM^[20a]^ were considered but because 5E8M is in the apo form and a conformational change is observed in the heparanase structure in 6ZDM when in complex with **3**, they were deemed not suitable.

**Figure 5:**
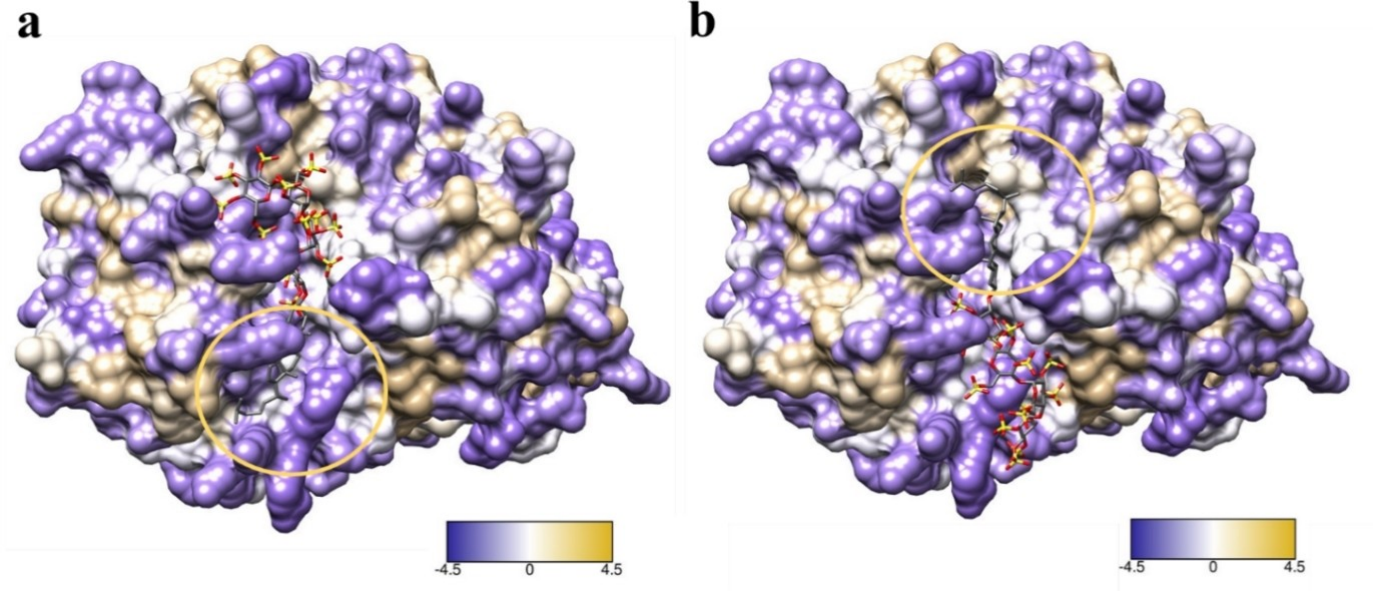
Conformations *a* and *b* were selected for simulations based on the orientation of cholestanol towards the hydrophobic regions (circled in yellow) and the carbohydrate chain covering the catalytic pocket. Purple indicates hydrophilic and white tan and circle shows hydrophobic surface.

Fondaparinux (**4**) was firstly docked into heparanase using SeeSAR^[23]^ version 10 and shown to span both the HBD‐1 and HBD‐2 binding region. The active site was defined based on the co‐crystallised ligand (HS tetrasaccharide) and the structure of fondaparinux was taken from crystal structure 4R9W. Fondaparinux residues fit into subsites −2 to +3 (numbered from non‐reducing to reducing end of the chain^[24]^) where β‐GlcA and α‐GlcNS3S6S sit at the −1 and +1 subsites, respectively. This linkage is shown to reside in proximity to the heparanase cleavage site near the catalytic residues Glu225 and Glu343. This model fits with the literature that heparanase cleaves fondaparinux at the β‐GlcA‐(1→4)‐α‐GlcNS3S6S linkage^[22]^ and is in agreement with the reported docking of fondaparinux with heparanase.^[11a]^ Attempts to similarly dock PG545 using SeeSAR were unsuccessful because SeeSAR cannot dock ligands on the surface. PG545 was therefore docked using GlycoTorch Vina,^[25]^ a ligand−protein docking program specifically developed for HS−protein interactions, which provided a ligand−protein complex. Several ligand−protein complexes of different conformations were obtained on docking from which two conformations were selected which blocked the catalytic residues (Glu225 and 343) essential for cleaving substrate (Figure 5). The two conformations selected were based on the most populated clusters and the orientation of cholestanol towards the hydrophobic surface present near the catalytic pocket (shown in Figure 5, circled with cholestanol).

**Figure 6:**
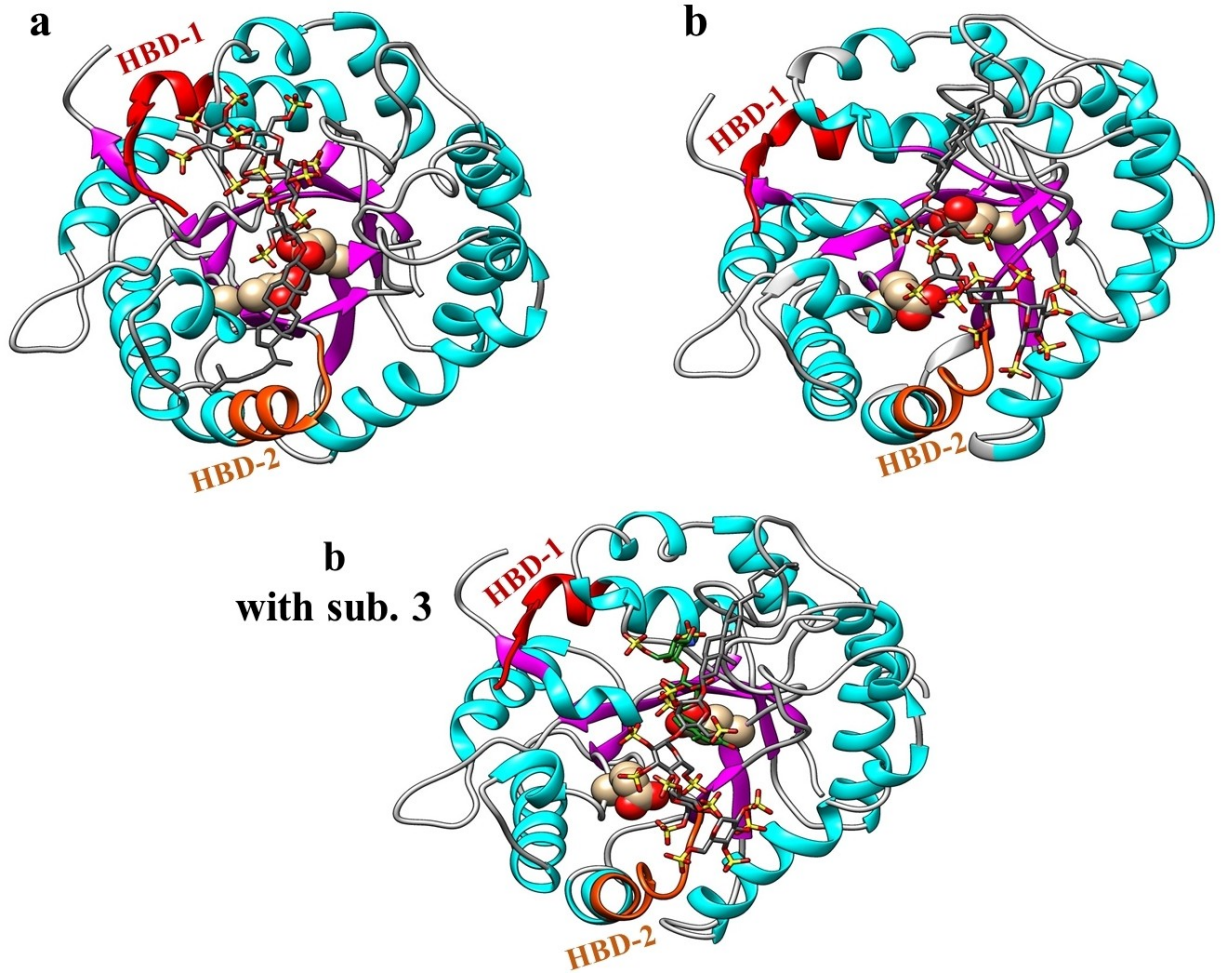
Representative conformations obtained from the most populated cluster of the MD simulations of the PG545−HPSE complexes are shown. Glu 225 and 343 in conformation *a* and *b* are shown as spheres in the catalytic pocket covered by PG545. HBD‐1 (residues 158–171) and HBD‐2 (residues 270–280) are shown in red and orange, respectively. Conformation *b* was also subjected to MD in the presence of substrate **3** (green) to investigate the partial inhibition of hydrolysis by PG545. Average interactions obtained from the MD trajectories are shown in Figure S8. Hydrogens of PG545 and C‐terminal of heparanase are not shown for clarity.

The two conformations also cover the catalytic pocket and lie in the HBD‐1 and HBD‐2 regions. PG545 interactions for conformations *a* and *b* with key heparanase residues present near the catalytic pocket were obtained using Discovery Studio and are shown in Figure S7. Sulfates in conformation *a* show electrostatic interactions with Asp 62, Asn 64, Asp 68, Thr 97, Lys 98, HBD‐1 residues (Lys 159, Phe 160, Lys 161), Lys 231, HBD‐2 residues (Arg 272, Lys 274), Arg 303, Tyr 348, Gly 350, Gln 383, Ala 388, and Gly 389. Cholestanol in conformation *a* shows hydrophobic interactions with Lys 231, Arg 272, Lys 274, and Tyr 348. Similarly, sulfates in conformation *b* show electrostatic interactions with Thr 97, Glu 225, Lys 231, Lys 232, HBD‐2 residues (Gly 269, Gln 270, Arg 272, Arg 273), Arg 303, Lys 325, Tyr 348, and Ala 388. Cholestanol in conformation *b* shows hydrophobic interactions with Ala 388. The two ligand−protein complexes were then subjected to MD simulations to understand the interactions and dynamics of PG545 in complex with heparanase. Figure 6 shows representative poses of the most populated cluster obtained from the MD trajectory of each system. The simulations of both conformations demonstrate that negatively charged sulfates on PG545 interact with positively charged residues on the heparanase surface and that the cholestanol tends to stay oriented towards the hydrophobic pocket. The latter help PG545 reside above the catalytic pocket (Figure 6) to block the catalytic residues (Glu225 and 343). The binding free energies (Δ*G*) of the two complexes were calculated using MM−GBSA which provided the energy components such as Δ*E*_VDW,_ Δ*E*_electro,_ Δ*G*_solv._ and Δ*G*_total_ energy (see Table 2).

**Table 2:** Predicted relative MM−GBSA free energies (kcal mol^−1^) and individual energy terms for selected conformations of PG545−HPSE complex.

Conformations	Δ*E*_VDW_	Δ*E*_electro_	Δ*G*_solv._	Δ*G*_total_
conformation *a*	−56.4±7.2	−3502.3±409.5	3509.7±402.1	−49.0±15.9
conformation *b*	−67.1±6.87	−4121.9±155.9	4117.6±148.9	−71.5±12.4
conformation *b* w/sub **3**	−57.9±6.2	−3481.9±108.0	3450±102.2	−89.2±14.3

*All energies are in (kcal/mol); calculated binding energies do not include entropy term and are averaged from a single run. All values consider relative free energies of binding ± Std deviation.

It was determined that conformations *a* and *b* were stable having free binding energy (Δ*G*_total_) of −49.0±15.9 kcal/mol, and −71.5±12.4 kcal/mol for conformations *a* and *b*, respectively. Further MD simulations were carried out for PG545 in the presence of disaccharide substrate **3** to investigate the partial inhibition of hydrolysis of **3** by PG545. An equilibrated HPSE−PG545 complex was taken of conformation *b* after 50 ns of MD simulations and **3** was added by superimposition using UCSF Chimera. Conformation *a* in complex with **3** was not subjected to MD simulations because of a steric clash between PG545 and **3** after addition to the PG545−HPSE complex. The new complex built of conformation *b* was subjected to MD simulations and the affinity of PG545 was measured in the presence of **3**. The free energy values obtained from PG545−substrate−HPSE complex (conformation *b* with substrate **3**) showed that the presence of substrate does not affect the affinity of PG545 towards heparanase (Table 2). From MD simulations, it was observed that **3** stays in the catalytic pocket with GlcA at −1 subsite (as reported)^[20a]^ even in the presence of PG545 (Figure 6). The running averages of root mean square deviation (RMSD) for all three simulated systems are shown in Figure S9. The simulations of all three systems show minor RMSD from the docked conformation of the complex. This suggests that the protein doesn't undergo significant conformational change upon binding of the ligands.

It was also observed in conformation *b* without substrate that the carbohydrate domain of PG545 tends to occupy the HBD‐2 region and cholestanol occupies the hydrophobic pocket towards HBD‐1. This covers most of the active site over the catalytic pocket but the cholestanol shows flexibility during simulation, which might allow partial access to small substrates like **3** to enter, in line with the partial inhibition data obtained above. PG545 has previously been shown to potently inhibit the hydrolysis of fondaparinux (**4**) with *K*_i_ of ∼6 nM,[^13^, ^18^] implying that it completely blocks the large pentasaccharide substrate from accessing the active site. Further, fondaparinux was docked using the 4E9C crystal structure using SeeSar (Figure S10) and was overlaid with substrate **3**. This showed that the pentasaccharide **4** covers both HBD‐1 and HBD‐2 binding regions whereas the smaller substrate **3** occupies only the HBD‐1 binding region. These findings are consistent with the notion that PG545 is able to block fondaparinux binding to the active site of heparanase but is unable to inhibit a smaller substrate like **3** to the same extent.

### Interaction energy analysis pinpoints important heparanase−PG545 contacts

PG545 was next analysed for its electrostatic and van der Waals’ interactions with heparanase residues. The PG545−heparanase complexes obtained from the MD simulations with and without substrate **3** were used to calculate the pairwise per‐residue decomposition energy between PG545 and heparanase residues. Figure S8 shows the electrostatic interactions of sulfates on PG545 with positively charged heparanase residues and van der Waals interactions of cholestanol with hydrophobic residues. Conformation‐wise analysis of interactions was carried out using conformation *a* in the absence of **3** (Figure S8A) and conformation *b* in the absence (Figure S8B) and presence (Figure S8C) of **3**, and their interactions with heparanase demonstrate that PG545 binds to heparanase on the surface covering the catalytic site and makes electrostatic and van der Waals interactions with the key residues in HBD‐1 and HBD‐2 region and other residues around the catalytic pocket. To summarise, residues 270–275 (Gln−Pro−Arg−Arg−Lys−Thr) from HBD‐1 and residues 159–161 (Lys−Phe−Lys) from HBD‐2 along with other residues such as Lys 98, Ser 226, Asn 227, Lys 231, Lys 232, Asn 301, Arg 303, Lys 325 and Tyr 348 are the most crucial residues for PG545 binding through electrostatic interactions. The most crucial for van der Waals interactions with cholestanol are: Phe 160, Glu 225, Ser 228, Lys 231, Lys 232, Gln 270, Pro 271, Arg 272, Pro 287, Asn 301, Gly 302, Arg 303, Tyr 348, Gly 349, Gly 350, Gly 351, Ala 352, Pro 353, Asn 390.

## Conclusion

In summary, structural analysis of PG545 using 1D and 2D NMR experiments was carried out where intra‐, and inter‐residue H−H interactions were obtained by selectively irradiating the anomeric proton of each sugar residue using one‐dimensional selective versions of the NOESY and TOCSY which were also analysed in combination with two‐dimensional ^1^H‐NOESY, ^1^H ^13^C‐HSQC‐NOESY spectra. The selective 1D TOCSY and NOESY spectrum of each residue confirmed the intra‐ and inter‐residual connectivity. Further, intra‐ and inter‐residue H−H distances were calculated on the basis of the NOEs measured from the NMR spectra and MD simulations of PG545 and the calculations from both sources were in agreement. The data also confirmed the occurrence of a skew boat‐like conformation intermediate between ^3,O^ *B* and ^3^ *S*_1_ for residue I at the reducing end which is not present in **2** (non‐sulfated PG545). PG545 was also studied for its interactions with heparanase by molecular docking and MD simulations. Docking provided several ligand−protein complexes of which two conformations were selected having the cholestanol oriented towards different hydrophobic patches near the catalytic pocket and with the carbohydrate domain covering the active site residues (Glu225 and 343). The ligand−protein complexes selected were then subjected to MD simulations to demonstrate the interaction and stability of PG545 in complex with heparanase. Free binding energies (Δ*G*) of the complexes were calculated which showed that the cholestanol plays a crucial role in PG545 binding together with electrostatic interactions of the sulfated sugar backbone. MD simulations of PG545−heparanase and docking of fondaparinux **4** was also performed in the presence of disaccharide substrate **3**. The simulations showed that the cholestanol side chain is flexible and allows partial access to the active site for small molecules such as substrate **3**, but completely blocks the longer substrate **4**. This suggests that smaller substrates such as **3** are not ideal for assaying heparanase inhibitors of the PG545 structural class. Such inhibitors are best assayed using **4** or larger substrates.^[22]^ Detailed PG545−heparanase interaction analysis was carried out by calculating the per‐residue pairwise energies and plotting the pairwise per‐residue electrostatic and van der Waals’ interactions. Interactions of all PG545−heparanase conformations with and without substrate **3** demonstrate that PG545 binds to heparanase on the surface covering the catalytic site and it makes electrostatic and van der Waals interactions with key residues in the HBD‐1 and HBD‐2 regions and other residues around the catalytic pocket. The hydrophobic regions available near the catalytic pocket are crucial for the binding of the cholestanol side chain of PG545. This study will further aid in developing next generation substrates and inhibitors of heparanase with improved efficacy.

## Conflict of interest

The authors declare no conflict of interest.

1

## Supporting information

As a service to our authors and readers, this journal provides supporting information supplied by the authors. Such materials are peer reviewed and may be re‐organized for online delivery, but are not copy‐edited or typeset. Technical support issues arising from supporting information (other than missing files) should be addressed to the authors.

Supporting Information

## Data Availability

The data that support the findings of this study are available from the corresponding author upon reasonable request.
