# Supplementary material for: Structural Insights into Pixatimod (PG545) Inhibition of Heparanase, a Key Enzyme in Cancer and Viral Infections
Source: Chemistry. 2022 Jan 31;28(11):e202104222. doi: 10.1002/chem.202104222 (PMC9303737; doi:10.1002/chem.202104222)

Chemistry–A European Journal

Supporting Information

Structural Insights into Pixatimod (PG545) Inhibition of Heparanase, a Key Enzyme in Cancer and Viral Infections

Mohit Chhabra, Jennifer C. Wilson,* Liang Wu, Gideon J. Davies, Neha S. Gandhi,* and Vito Ferro*

1. Experimental
    a. NMR Spectroscopy S2
    b. Molecular Docking S2
    c. Molecular Dynamics S3
    d. Relative free energy of binding calculations S5
    e. Analysis and visualisation S7
    f. Heparanase inhibition assay S8
    g. References S9
2. Figures S1-S10, Table S1 S11-S22

Experimental

NMR Spectroscopy

PG545 (5 mg), prepared as previously described,[1] was dissolved in D_2O (240 μL) under a nitrogen atmosphere and ^1H, 1D-NOESY (mixing times = 500 ms and 800 ms), and TOCSY (mixing time = 180 ms) spectra were recorded at 298 K on a Bruker Avance spectrometer operating at 600 MHz. All the chemical shifts are reported in ppm and are referenced to internal water (^1H 4.79 ppm). 2D ^1H-NOESY, 2D ^1H ^{13}C-HSQC-NOESY spectra of PG545 were acquired with 80 scans and a minimum of 512 slices at 298 K and 600 MHz with NOESY mixing times of 500 ms and 800 ms. NOEs obtained from the spectrum (500 ms) were integrated using the standard Bruker (TopSpin version 3.6.2) software and were categorized using the distance constraints: 1.8–2.8 Å (strong), 1.8–3.3 Å (medium) and 1.8–5.0 Å (weak). The rNOE distance is calculated from the peak integral I using equation rNOE = r_{ref} $(I_{ref}/I)^{1/6}$ with a reference distance (r_{ref}) of 2.5 Å between H1 and H2 protons of glucose. Calculated rNOE distances for composite NOEs are "virtual distances" that are shorter than any of the "real distances" between the contributing H-H pairs.

Molecular Docking

The crystal structures of heparanase (PDB ID: 5E9C and 6ZDM) were compared for any conformational changes before docking and MD simulations. The 5E9C crystal structure cocrystallised with heparin tetrasaccharide,[2] was selected for docking studies. Water and all non-standard residues were removed before docking, and Glu225 was manually protonated. The structure of fondaparinux **4** (PDB ID: 4R9W) was firstly docked with heparanase. Docking of fondaparinux was performed using SeeSAR version 10,[3] a software package available for docking and optimisation of ligands that bind in a well-defined pocket. The active site was defined based on the co-crystallised ligand (heparin tetrasaccharide) with heparanase. For each

fondaparinux, 10 new "docked conformations" were generated, and all were evaluated with the built-in hydrogen dehydration (HYDE) scoring function. The conformation was chosen based on superimposition of disaccharide segment (GlcNS6S-GlcA) of fondaparinux with GlcNS6S-IdoA segment of heparin tetrasaccharide for further analysis.

Glycam carbohydrate builder (Glycam.org)[4] was used to build the sulfated tetrasaccharide backbone of PG545, which was then modified by adding a cholestanol group at the reducing end using Discovery Studio,[5] to obtain the required structure. A centroid was selected of the heparin-binding region from the crystal structure, which was used to define the coordinates for the docking of PG545. The docking of PG545 was performed using coordinates -16.3, 12.95, 58.6 respectively within the grid box size of 40 × 40 × 40 Å around the co-crystallised ligand (heparin). PG545 was docked using GlycoTorch Vina[6] with energy range, exhaustiveness and num_modes values set to be 12, 24, and 100, respectively. The conformations with the best-estimated affinities and cholestanol oriented towards different hydrophobic pockets were chosen for further analysis.

Molecular dynamics

The coordinates of protein and the ligand conformations were chosen from the two clusters of different conformations obtained from molecular docking and were used for MD simulations. AMBER ff14SB-ildn[7] force field was used for modelling the protein, and Glycam06[8] (version j) was employed for sugars. Lipid14[9] was used for cholestanol, and the remaining parameters were taken from GAFF.[10] The input files for MD were prepared using cpptraj of AmberTools18[11] and MD simulations were carried out using the Amber16 software package.[12] The systems were solvated using TIP3P[13] water molecules in a truncated octagonal box, having a 12 Å distance between the solute and the wall of the box. An

appropriate number of sodium (Na^+) or chloride (Cl^-) ions were added to neutralise the system followed by addition of Mg^{2+} and Cl^- to get the final concentration of 0.1M of $MgCl_2$. The partial atomic charges of the carbohydrate backbone and aglycon (cholestanol) were derived using the Glycam06[8] and restrained electrostatic potential (RESP) charge fitting methodology,[14] respectively. RESP was computed at the HF/6-31G∗ level of theory and obtained from RED server.[15] The charges for the aglycon were derived separately, and the total charge on the aglycon was set to be the same as the charge on the anomeric hydroxyl groups of sugar from Glycam, i.e., -0.194, which makes the compound neutral as required for simulations. Energy minimization was performed in five steps, four of them using 3000 cycles of steepest descent and 5000 cycles of conjugate gradients for each one; the heavy atoms were restrained by a harmonic potential of 100 kcal/mol*$Å^2$. In the last step, 5000 cycles of steepest descent and 10 000 cycles of conjugate gradients were performed with no restraints. The heating and equilibration stage was divided into 2 steps. A position-restrained phase of MD simulations was carried out for 500 ps by first slowly heating up the systems from 0 to 300 K for 100 ps and then maintaining the temperature at 300 K for another 400 ps in the NVT ensemble. During this phase, a soft-force constraint (10 kcal/mol $Å^2$) was applied to restrain the complexes. After that, a 2 ns equilibration phase was employed in an NPT ensemble. The SHAKE algorithm[16] was used to restrain the bonds involving hydrogens and the particle-mesh Ewald (PME) method[17] was used to estimate the long-range electrostatic interactions. A cut off distance of 12 Å was applied to compute the non-bonded interactions and the temperature was maintained at 300 K using the Langevin thermostat[18] with a collision frequency of 5 ps^{-1} . The equilibration and production MD simulation runs for the ligand-protein complexes were carried out for 50 ns and 350 ns, respectively. The frames in the trajectories were saved every 10 ps. Periodic boundary conditions were applied throughout the simulations. The RMSD of each simulation was calculated using mainchain atoms (N, Cα, C, O) of the heparanase

enzyme. Trajectories for all three systems were grouped into 5 clusters using the k-means clustering algorithm in cpptraj of AmberTools18. The mainchain atoms and Cβ of the heparanase were used for clustering. The top (i.e., most populated) cluster was selected for analysis to include > 20% of the trajectory.

PG545 was simulated alone in a solvent system containing water to investigate the conformational changes. Glycam carbohydrate builder was used to build the sugar backbone and then a cholestanol group was added at the reducing end using Discovery Studio. The systems were solvated using TIP3P water molecules in a truncated octagonal box, having a 15 Å distance between the solute and the wall of the box. Ions were only added to neutralise the net charge of the system. The equilibration and production MD simulation runs were carried out for 50 ns and 400 ns, respectively using the MD protocol described above.

Relative free energy of binding calculations

All trajectories were analysed using the cpptraj[19] module of AmberTools18.[11] Molecular Mechanics /Generalized Born surface area (MM-GBSA) was performed on the last 35000 complex frames (150 ns) to calculate the relative free binding energy of PG545-HPSE complexes using the one-trajectory approximation. The linear combinations of pairwise overlaps 'LCPO' was used to calculate solvent accessible surface areas[20] whereas the polar component of the desolvation energy was determined via Onufriev's GB (igb = 5). Entropy calculations were not carried out due to the expensive computational cost of running a big complex. Calculations were performed with a salt concentration of 0.1 M, GB model igb = 5 and the PBR radii was set to mbondi2. All the units are reported in kcal mol^{-1} for van der Waals, electrostatic and total free energy.

The free binding energy ($\Delta G_{binding}$) for MM-GBSA[21] is calculated by using the thermodynamic free energy equation; where ΔH is enthalpy change and T and ΔS are temperature and change in entropy of the system, Eq. (1)

$$\Delta G_{binding} = \Delta H - T\Delta S \qquad (1)$$

Free energies of complex, unbound protein and ligand molecular system are calculated as equation (2) below where E_{MM} is total molecular mechanics energy of system in the gas phase, G_{solv} is solvation free energy, T is temperature and S is the entropy of system; Eq. (2).

$$\Delta G_{binding} = \Delta E_{MM} - \Delta G_{solv} - T\Delta S \qquad (2)$$

E_{MM} (molecular mechanics energy in the gas phase) is calculated as the sum of bonded energies (angle bending, dihedral torsional rotation and bond stretching) and non-bonded energies (electrostatic interactions and Van der Waals); Eq. (3).

$$\Delta E_{MM} = \Delta E_{bonded} + \Delta E_{elec.} + \Delta E_{VDW} \qquad (3)$$

G_{solv} (solvation energy) constitutes both polar and non-polar contributions where polar is accounted for generalized Born model and the non-polar is proportional to solvent-accessible surface area (SASA) according to LCPO algorithm, Eq. (4).

$$\Delta G_{solv} = \Delta G_{PB/GB} + \Delta G_{SASA} \qquad (4)$$

To obtain more detailed information about the contribution of binding residues, the MM/GBSA module of AmberTools18 was used to compute the pairwise per-residue energy decompositions for every individual frame, adding 1 to 4 energy terms to internal energy terms; the energies for every residue were averaged over the last 35000 frames (150 ns) of production dynamics. The outer dielectric and solute dielectric constants were set to 80 and 1, respectively.

Decomposition energy schemes were developed by Gohlke and co-workers[22] and energy terms are calculated using the following equation (5).

$$\Delta E_{Decomp} = \sum_{\substack{j \in complex \\ j \in receptor}} \left(E_{Complex}(i,j) - E_{Receptor}(i,j) \right)$$

$$+ \sum_{\substack{j \in complex \\ j \in ligand}} \left(E_{Complex}(i,j) - E_{Ligand}(i,j) \right) \qquad (5)$$

Where the first and second terms are the average contribution over the snapshots, i, from MD simulations in residues j on the receptor and ligand, respectively. The $E_{GBTOTAL}(i,j)$ is a contribution of gas phase and solvation energies (equation 6,7);

$$E_{GBTotal}(i,j) = E_{Gas}(i,j) + E_{GBSolv}(i,j) \qquad (6)$$

$$= E_{VDW}(i,j) + E_{ELE}(i,j) + E_{GB}(i,j) + E_{Surf}(i,j) \qquad (7)$$

The per-residue decomposition energy includes nonbonded electrostatic energy (ΔE_{ELE}), VDW energy (ΔE_{VDW}), polar solvation energy (ΔE_{GB}) from the generalized Born model, and the nonpolar energy. Entropy is not included in the decomposition method.

Analysis and visualisation

The glycosidic torsions were defined as: φ = H1-C1-O4-C4, ψ = C1-O4-C4-H4 and ω = O5-C5-C6-O6. The relative free energy surfaces corresponding to the glycosidic angles were computed using the expression ΔGx = −kBT ln(ρx), where kB is the Boltzmann constant, T is

the temperature, and ρx is the probability density of the geometric coordinate x. Generalised pseudorotation coordinates by Cremer and Pople were adopted to estimate the puckering conformation of pyranose rings.[23] The conformational analysis of PG545 was carried out using Ramachandran plots for each glycosidic linkage. Two-dimensional (2D) free energy surfaces of φ/ψ glycosidic torsions were constructed using GNU plot (an open-source plotting tool) - where coloured density maps allowed the localization of the most populated ϕ/ψ states. Cpptraj was used to calculate NOEs and dihedral angles. All MD trajectories were visualised using UCSF Chimera[24] version 1.14. Hydrophobic surfaces were created using ChimeraX v. 3D heat maps for electrostatic and van der Waals interactions from pairwise per-residue decomposition energy were plotted using GraphPad Prism version 8.0.0.

Heparanase inhibition assay

Inhibition reactions were carried out in 10 μL volumes of assay buffer, containing 150 nM HPSE, 30 μM **3** and varying amounts of PG545. Reactions were incubated at 37 °C for 2 h, then immediately quenched with 10 μL 1 M Na_2CO_3 pH 10.3. Quenched reaction mixtures (15 μL) were transferred to a black 384 well plate, and 4MU fluorescence measured with a Clariostar microplate reader using the default 4MU fluorophore profile. Plots of inhibition vs PG545 concentration ([PG545]) were calculated by subtracting background fluorescence from no enzyme control wells, before normalizing hydrolysis rates at each inhibitor concentration (V_I) to the hydrolysis rate in the absence of inhibitor (V_0). An IC_{50} constant were calculated by fitting plots of V_I/V_0 vs [PG545] using nonlinear regression to the 4 parameter logistic curve function V_I/V_0= min+((max-min)/(1+([Hep]/IC_{50})^(-Hillslope)). K_i was estimated from IC_{50} using the Cheng-Prusoff approximation[25] K_i=IC_{50}/(1+[S]/K_M), using a previously estimated value of K_M.[26] Alternatively, plots of V_I/V_0 were fitted to the quadratic 'Morrison' equation for tight binding inhibitors: V_I=V_0*(1-(((([E]+[I]+Q)-((([E]+[I]+Q)^2)-

4*[E]*[I])^0.5))/(2*[E]))); Q=(K_I*(1+([S]/K_M))). Data were analyzed and plotted using Sigmaplot 13.0.

References

[1] M. Chhabra, N. Wimmer, Q. Q. He, V. Ferro, *Bioconjugate Chem.* **2021**, *32*, 2420-2431.

[2] L. Wu, C. M. Viola, A. M. Brzozowski, G. J. Davies, *Nat. Struct. Mol. Biol.* **2015**, *22*, 1016-1022.

[3] SeeSAR version 10.1; BioSolveIT GmbH, Sankt Augustin, Germany, 2020, www.biosolveit.de/SeeSAR.

[4] Woods Group, Complex Carbohydrate Research Center, University of Georgia, Athens, GA. (http://glycam.org).

[5] BIOVIA, Dassault Systèmes, Discovery Studio, San Diego: Dassault Systèmes, 2020.

[6] E. D. Boittier, J. M. Burns, N. S. Gandhi, V. Ferro, *J. Chem. Inf. Model.* **2020**, *60*, 6328-6343.

[7] J. A. Maier, C. Martinez, K. Kasavajhala, L. Wickstrom, K. E. Hauser, C. Simmerling, *J. Chem. Theory Comput.* **2015**, *11*, 3696-3713.

[8] K. N. Kirschner, A. B. Yongye, S. M. Tschampel, J. González-Outeiriño, C. R. Daniels, B. L. Foley, R. J. Woods, *J. Comput. Chem.* **2008**, *29*, 622-655.

[9] C. J. Dickson, B. D. Madej, Å. A. Skjevik, R. M. Betz, K. Teigen, I. R. Gould, R. C. Walker, *J. Chem. Theory Comput.* **2014**, *10*, 865-879.

[10] J. Wang, R. M. Wolf, J. W. Caldwell, P. A. Kollman, D. A. Case, *J. Comput. Chem.* **2004**, *25*, 1157-1174.

[11] D. A. Case, I. Y. Ben-Shalom, S. R. Brozell, D. S. Cerutti, T.E. Cheatham III, V. W. D. Cruzeiro, T. A. Darden, R. E. Duke, D. Ghoreishi, M. K. Gilson, H. Gohlke, A.W. Goetz, D. Greene, R Harris, N. Homeyer, Y. Huang, S. Izadi, A. Kovalenko, T. Kurtzman, T.S. Lee, S. LeGrand, P. Li, C. Lin, J. Liu, T. Luchko, R. Luo, D.J. Mermelstein, K.M. Merz, Y. Miao, G. Monard, C. Nguyen, H. Nguyen, I. Omelyan, A. Onufriev, F. Pan, R. Qi, D.R. Roe, A. Roitberg, C. Sagui, S. Schott-Verdugo, J. Shen, C.L. Simmerling, J. Smith, R. SalomonFerrer, J. Swails, R.C. Walker, J. Wang, H. Wei, R.M. Wolf, X. Wu, L. Xiao, D. M. York, P. A. Kollman in *AMBER 2018, University of California, San Francisco*.

[12] D. A. Case, R. M. Betz, D. S. Cerutti, T.E. Cheatham III, T. A. Darden, R. E. Duke, T. J. Giese, H. Gohlke, A. W. Goetz, N. Homeyer and S. Izadi in *AMBER 2016, University of California, San Francisco*.

[13] W. L. Jorgensen, J. Chandrasekhar, J. D. Madura, R. W. Impey, M. L. Klein, *J. Chem. Phys.* **1983**, *79*, 926-935.

[14] C. I. Bayly, P. Cieplak, W. Cornell, P. A. Kollman, *J. Phys. Chem.* **1993**, *97*, 10269-10280.

[15] a) F. Y. Dupradeau, A. Pigache, T. Zaffran, C. Savineau, R. Lelong, N. Grivel, D. Lelong, W. Rosanski, P. Cieplak, *Phys. Chem. Chem. Phys.* **2010**, *12*, 7821-7839; b) E. Vanquelef, S. Simon, G. Marquant, E. Garcia, G. Klimerak, J. C. Delepine, P. Cieplak, F.-Y. Dupradeau, *Nucleic Acids Res.* **2011**, *39*, W511-W517; c) F. Wang, J.-P. Becker, P. Cieplak, F.-Y. Dupradeau in *R.E.D. Python: Object oriented programming for Amber force fields, Université de Picardie - Jules Verne, Sanford Burnham Prebys Medical Discovery Institute, Nov. 2013*.

[16] J.-P. Ryckaert, G. Ciccotti, H. J. C. Berendsen, *J. Comput. Phys.* **1977**, *23*, 327-341.

[17] T. Darden, D. York, L. Pedersen, *J. Chem. Phys.* **1993**, *98*, 10089-10092.

[18] M. P. Allen, D. J. Tildesley, *Computer simulation of liquids*, Oxford University Press, DOI:10.1093/oso/9780198803195.001.0001, **2017**.

[19] D. R. Roe, T. E. Cheatham, *J. Chem. Theory Comput.* **2013**, *9*, 3084-3095.

[20] J. Weiser, P. S. Shenkin, W. C. Still, *J. Comput. Chem.* **1999**, *20*, 217-230.

[21] J. M. Hayes, G. Archontis *MM-GB(PB)SA calculations of protein-ligand binding free energies*, in *Molecular dynamics - Studies of synthetic and biological macromolecules*, (Ed. L. Wang), IntechOpen, **2012**, pp. 171-190.

[22] H. Gohlke, C. Kiel, D. A. Case, *J. Mol. Biol.* **2003**, *330*, 891-913.

[23] D. Cremer, J. A. Pople, *J. Am. Chem. Soc.* **1975**, *97*, 1354-1358.

[24] E. F. Pettersen, T. D. Goddard, C. C. Huang, G. S. Couch, D. M. Greenblatt, E. C. Meng, T. E. Ferrin, *J. Comput. Chem.* **2004**, *25*, 1605-1612.

[25] Y.-C. Cheng, W. H. Prusoff, *Biochem. Pharmacol.* **1973**, *22*, 3099-3108.

[26] L. Wu, N. Wimmer, G. J. Davies, V. Ferro, *Chem. Commun.* **2020**, *56*, 13780-13783.

Figure S1. A-C) *Top*: Overlaid ^{1}H, TOCSY, 1D-NOESY and TOCSY spectra of PG545 with selective irradiation of H1 of sugar residues II, III and IV (black letters - intra-residue proton of each residue, blue letters - inter-residue protons in 1D NOESY), all NOE's are marked in PG545 structure with blue * for NOE's and red * for irradiated proton. *Bottom*: 2D ^{1}H-^{13}C HSQC -TOCSY spectrum (blue) (600MHz, 298 K) overlaid with 1D NOE spectrum on selective irradiation of H1 of selected residue.

A) Selective irradiation of H1 of residue II

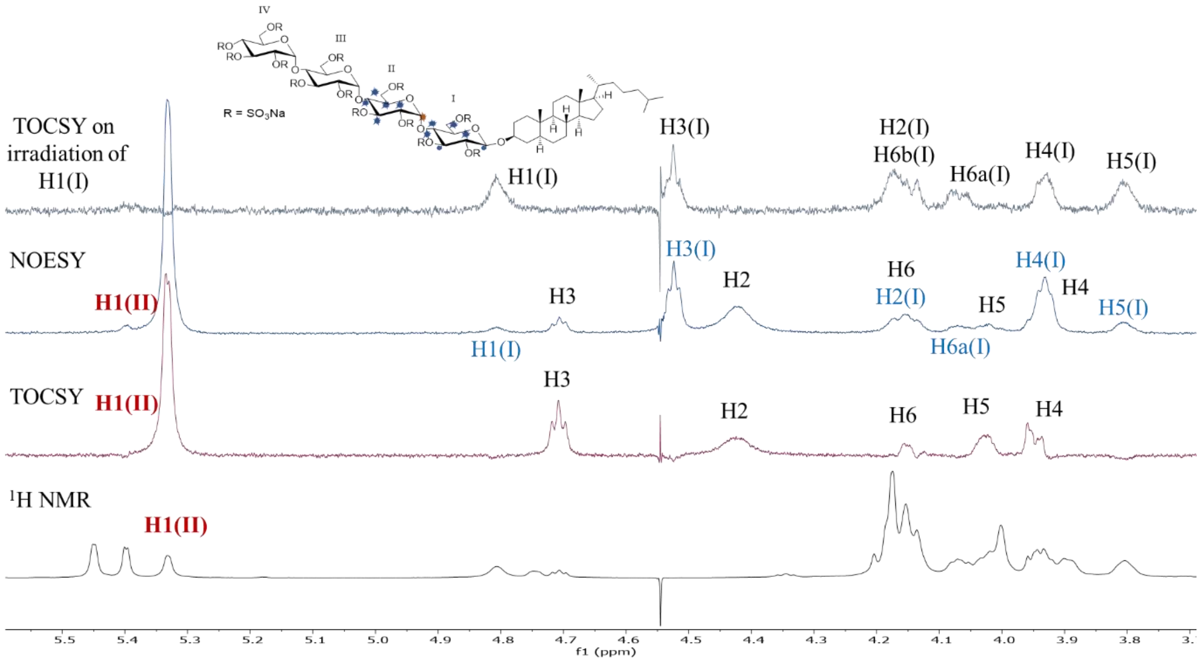

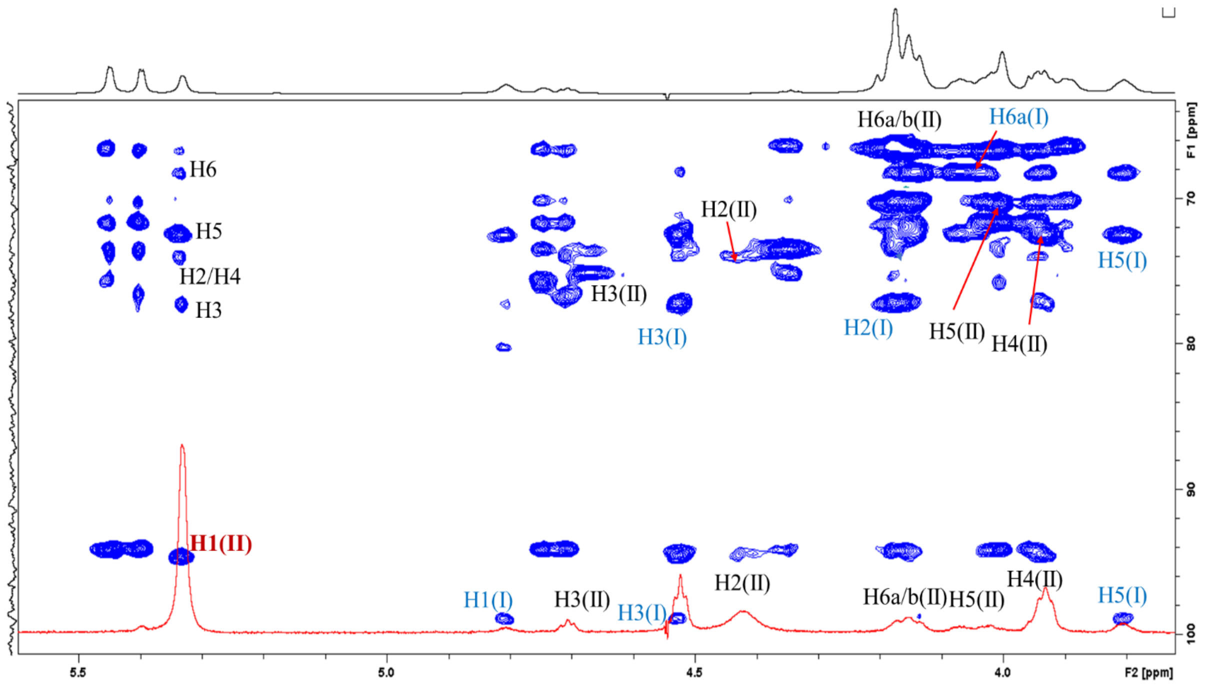

B) Selective irradiation of H1 of residue III

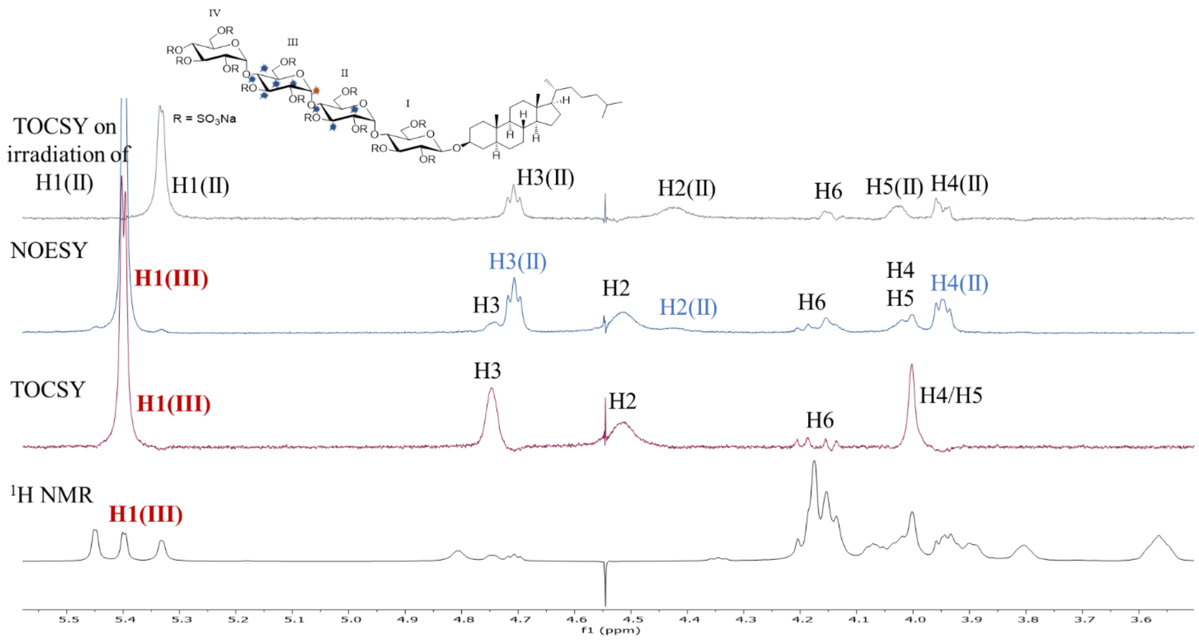

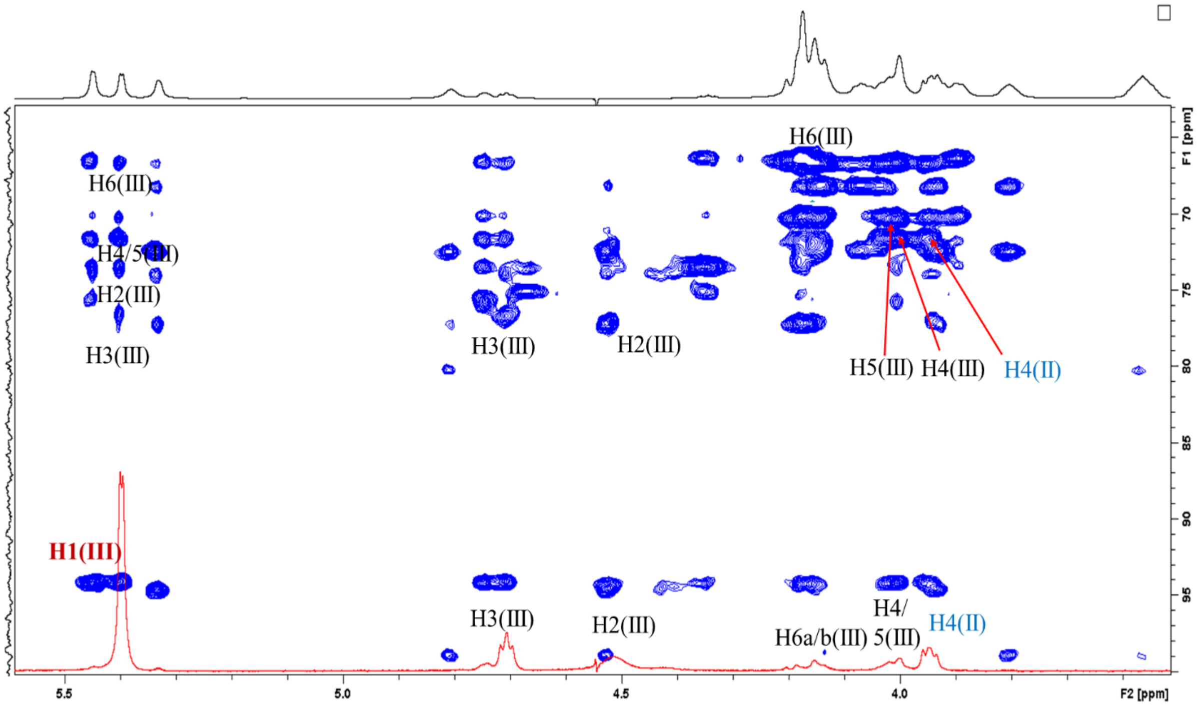

C) Selective irradiation of H1of residue IV

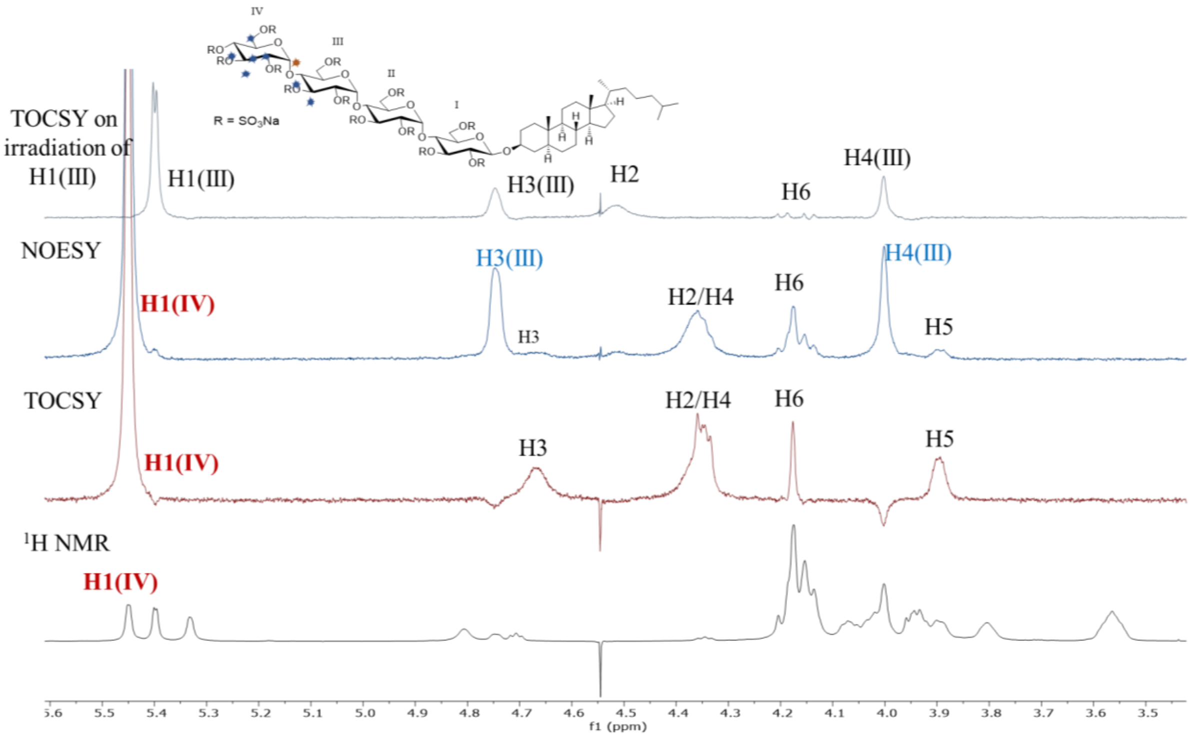

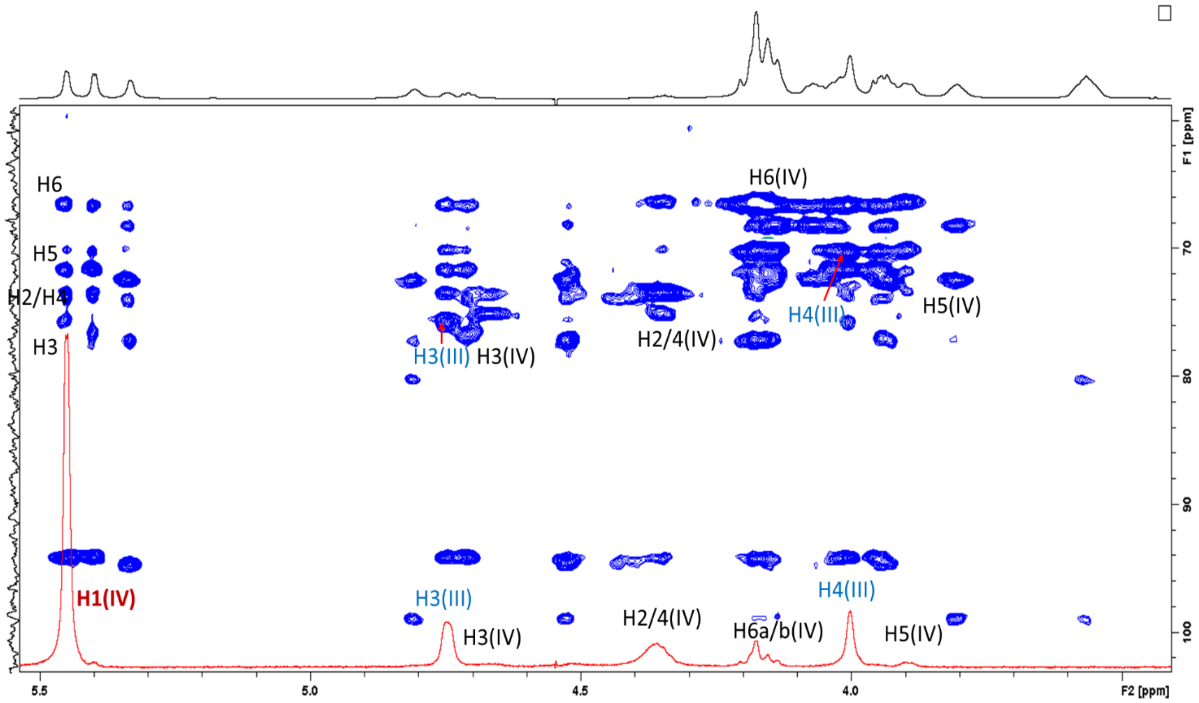

Figure S2. A) Superimposed 2D-NOESY and 1D-NOESY on irradiation of H3 of cholestanol (H-H NOE's in sky blue dots); B) ^{1}H-^{13}C HSQC (red, pink) ^{1}H-^{13}C HSQC -TOCSY spectrum (blue), aliphatic region and 1D selective TOCSY (green) spectrum with irradiation at H3 of cholestanol overlayed. These spectra allowed identification of the intra- and inter-residue crosspeaks.

A

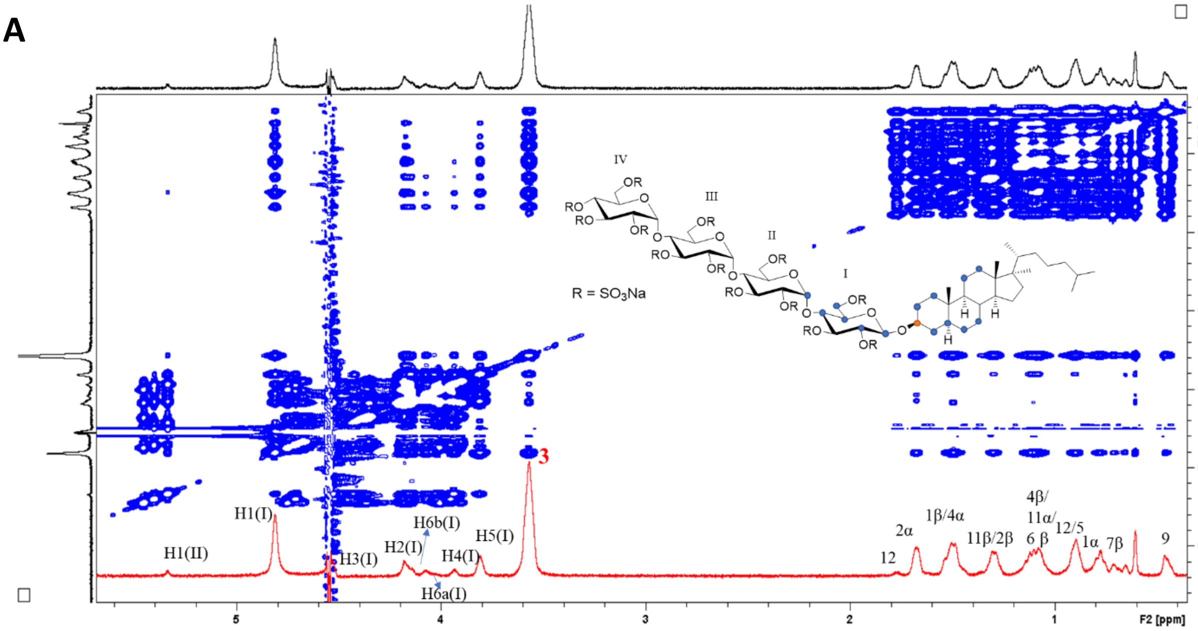

B

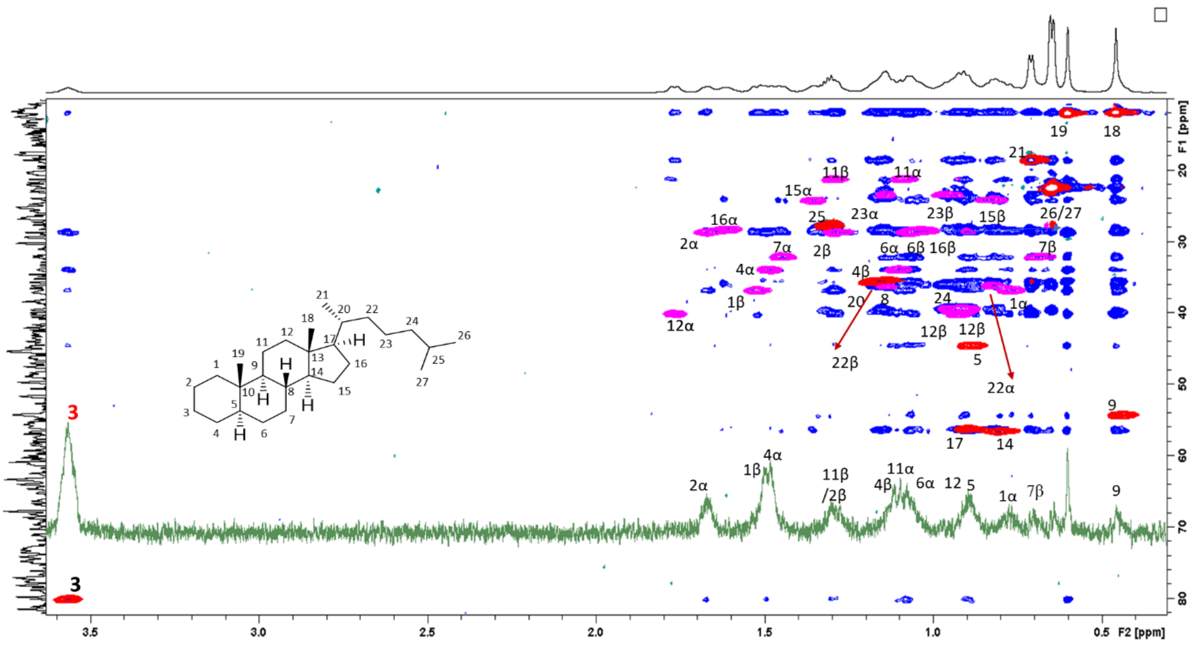

Figure S3. Glycosidic torsion angles φ = H1-C1-O4-C4, and ψ = C1-O4-C4-H4 of PG545 (**1**) and its non-sulfated precursor (**2**).

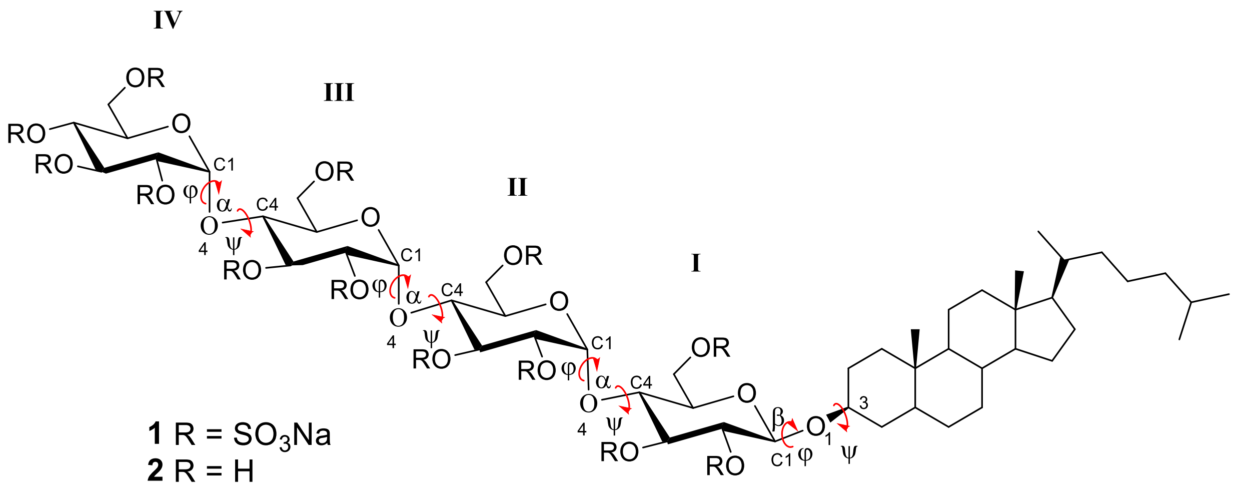

Table S1 φ /ψ angles of nonsulfated PG545 (**2**) and PG545 (**1**) from MD simulations.

Tetrasaccharide	Residue I (φ_1)	Residue II (φ_2)	Residue III (φ_3)	Residue IV (φ_4)
**2**	29.28 ± 49.70	-41.13 ± 17.15	39.90 ± 16.18	-39.92 ± 16.25
PG545	24.43 ± 46.00	-42.26 ± 12.87	-28.31 ± 39.76	-49.13 ± 17.55

Tetrasaccharide	Residue I (ψ_1)	Residue II (ψ_2)	Residue III (ψ_3)	Residue IV (ψ_4)
**2**	12.96 ± 40.59	-30.92 ± 14.61	-29.33 ± 15.06	-30.00 ± 14.50
PG545	12.70 ± 45.03	1.01 ± 30.41	-16.97 ± 31.93	-24.72 ± 18.44

Figure S4. Representative conformations of PG545 taken from MD simulations. Residue I adopts a skew boat-like conformation between $^{3,O}B$, $^{O}S_2$ and $^{3}S_1$; residues II and III are in $^{4}C_1$; residue IV flips between $^{4}C_1$ and $^{1}C_4$.

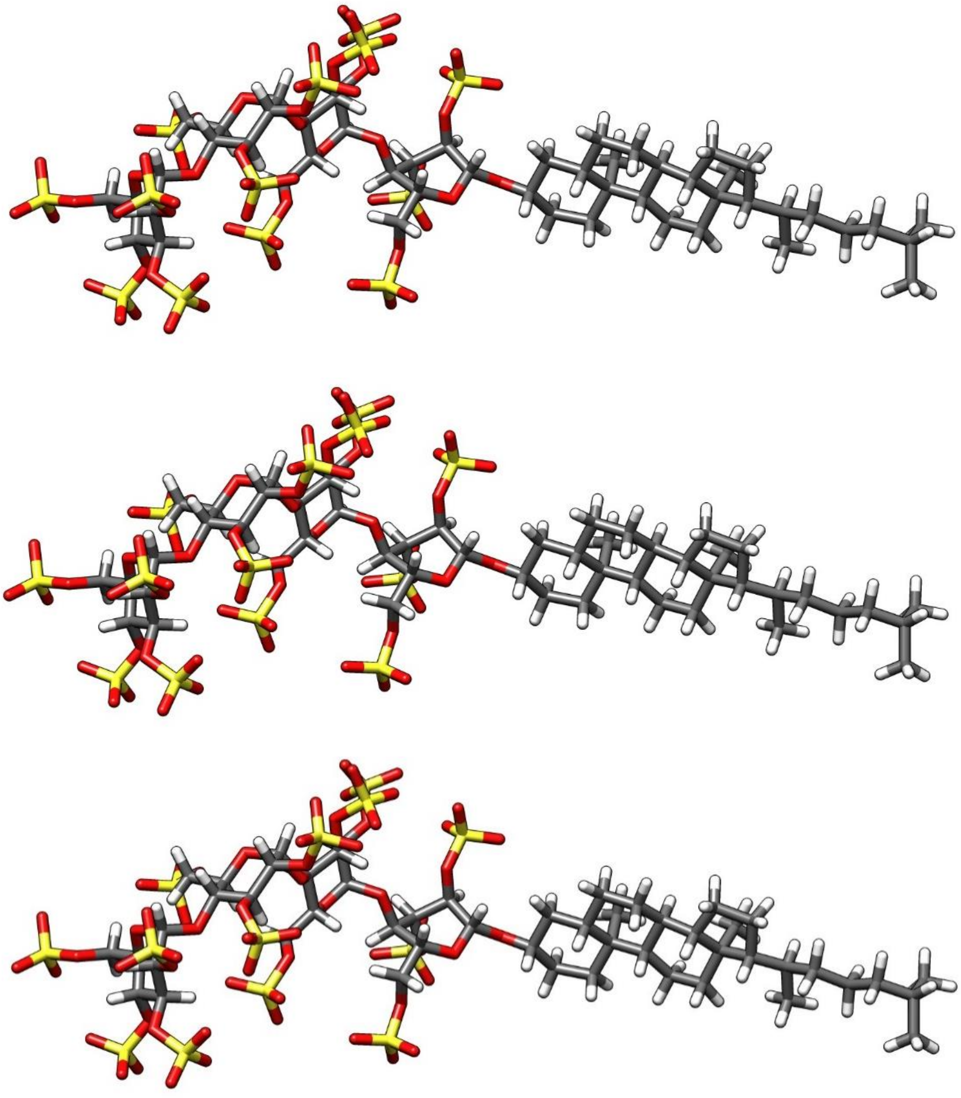

Figure S5. A) Average azimuthal angle (θ) plotted against simulation time to analyse the ring puckering of the sugar residues (I-IV). B) Zoom-in view of the meridian angle (*φ)* distribution for residue I, when θ = 90°.

A)

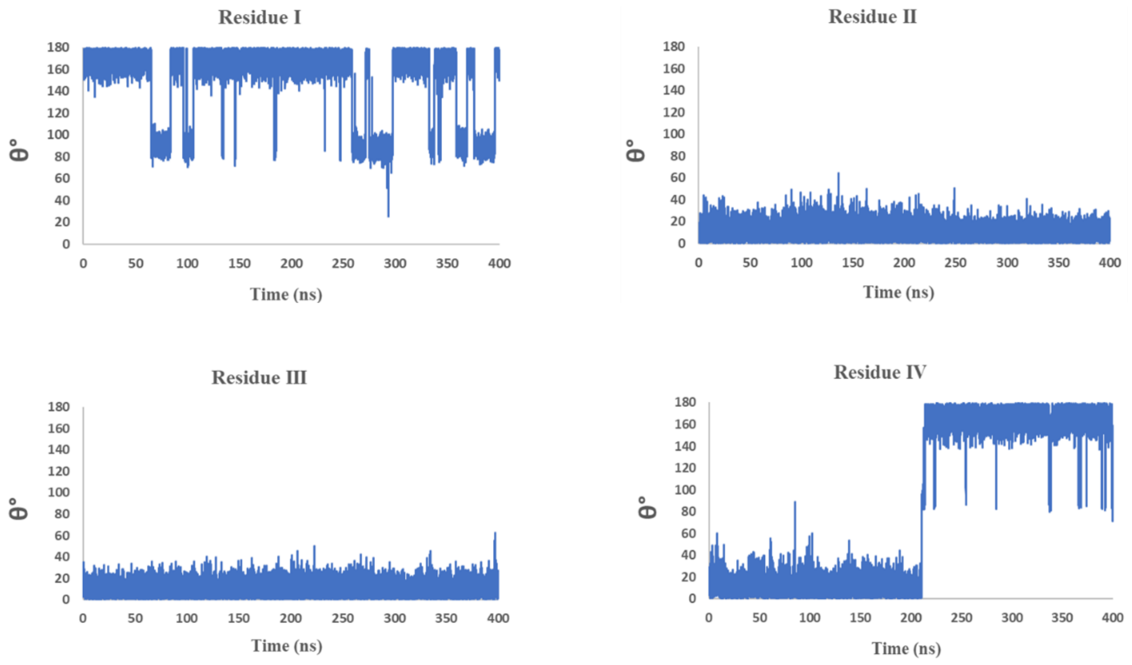

B)

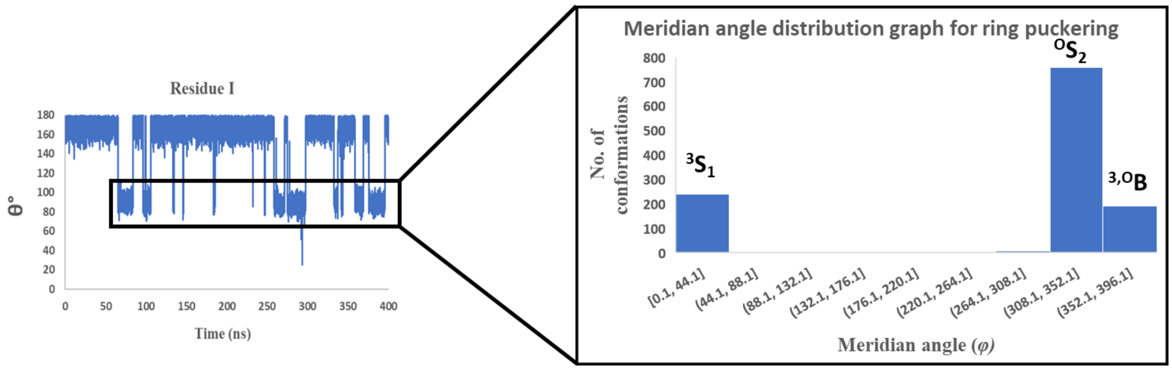

Figure S6. A. Inhibition of fluorogenic disacccharide **3** processing by HPSE in the presence of PG545. Estimated K_i is calculated from the IC_{50} using the Cheng-Prusoff approximation[25] and a K_M value of 10.3 μM for **3**. B. Estimated K_i calculated using the Morrison equation.[2,3]

A

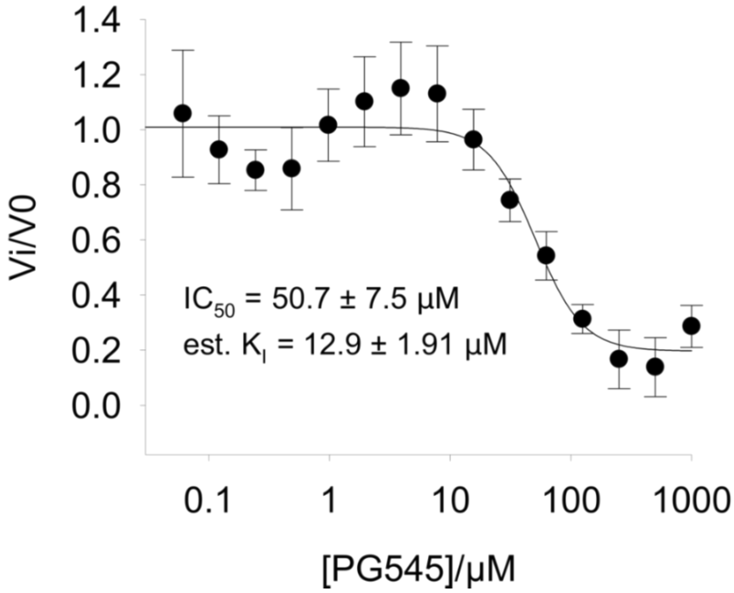

B

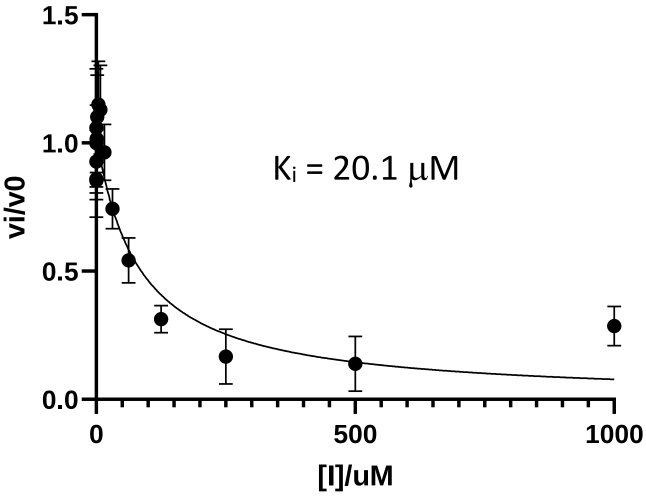

1) Cheng, Y.-C.; Prusoff, W. H., *Biochem. Pharmacol.* **1973,** *22*, 3099-3108.
2) Morrison, J. F., *Biochim. Biophys. Acta* **1969**, *185*, 269-286.
3) Copeland, R. A., *Methods Biochem. Anal.* **2005**, *46*, 1-265.

Figure S7. Electrostatic and van der Waals interactions of PG545 conformation **a** (top) and **b** (bottom) after docking with heparanase (PDB 5E9C). Key interactions with heparanase residues present near the catalytic pocket were obtained using Discovery Studio and are labelled in the figure. Green, orange, and pink (dashed lines) are used to show the classical and non-classical hydrogen bonds, and hydrophobic interactions, respectively.

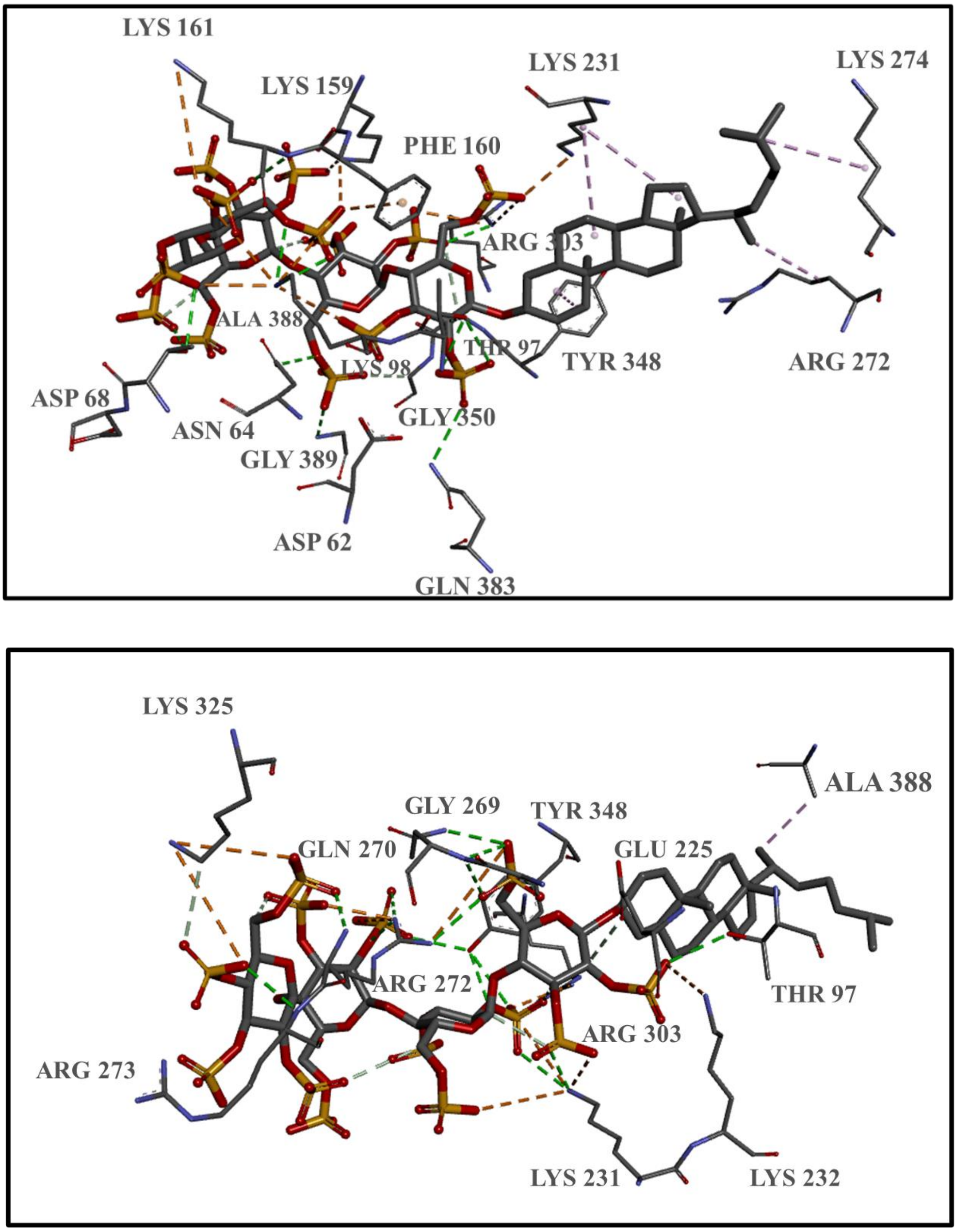

Figure S8. Electrostatic interactions (left) of PG545 sulfates and van der Waals interactions (right) of PG545 residues and cholestanol in conformation **a** and **b** observed with heparanase residues in the absence of substrate **3** are shown in plots (A-B). C shows the plots of PG545 (conformation **b**) interactions observed with heparanase residues in the presence of **3**. Protein residues with numbers are indicated on the x-axis and PG545 sulfates and **3** numbers are indicated on y-axis. ***Only interactions with amino acid residues present at a distance of ≤6Å around PG545 are listed here.

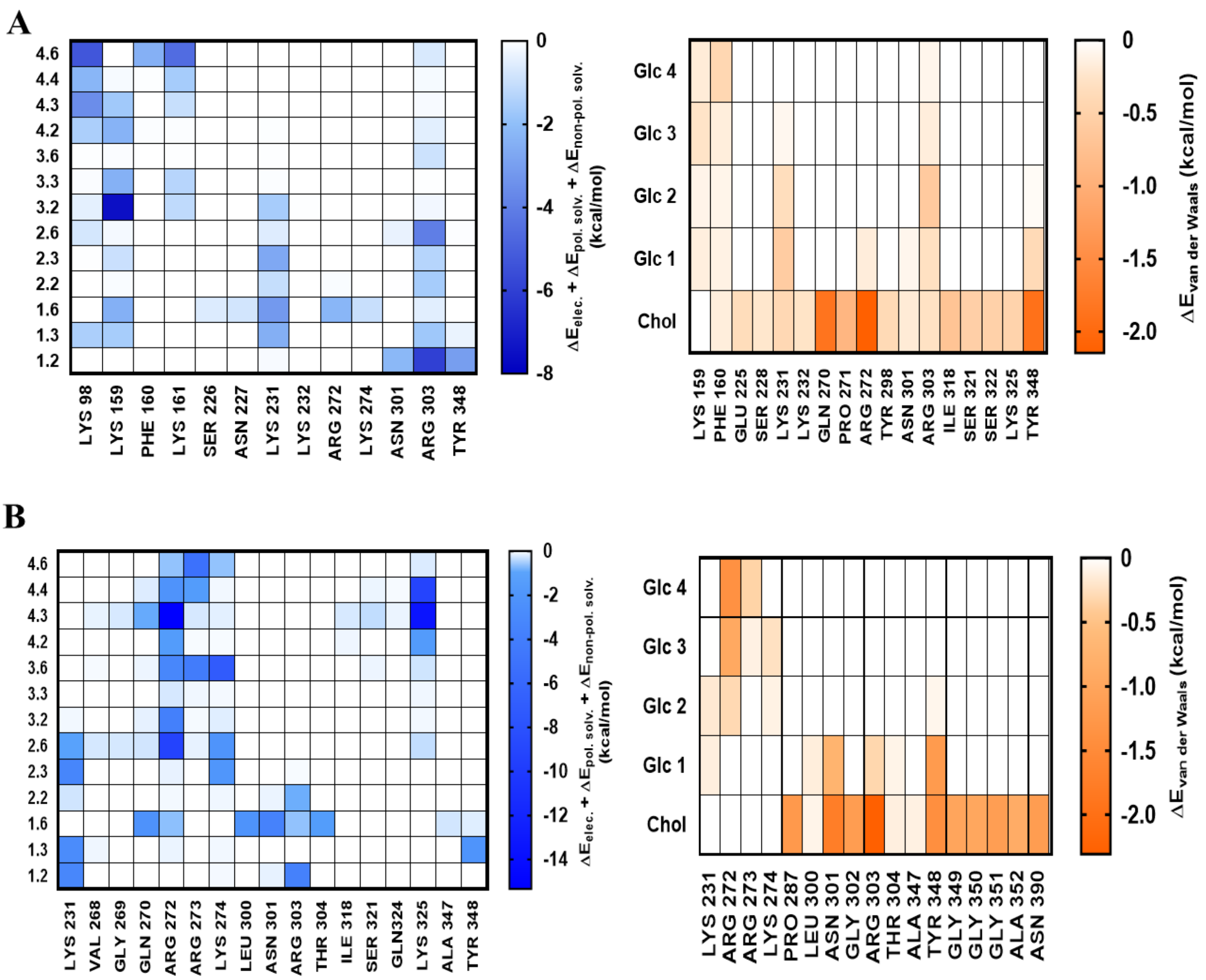

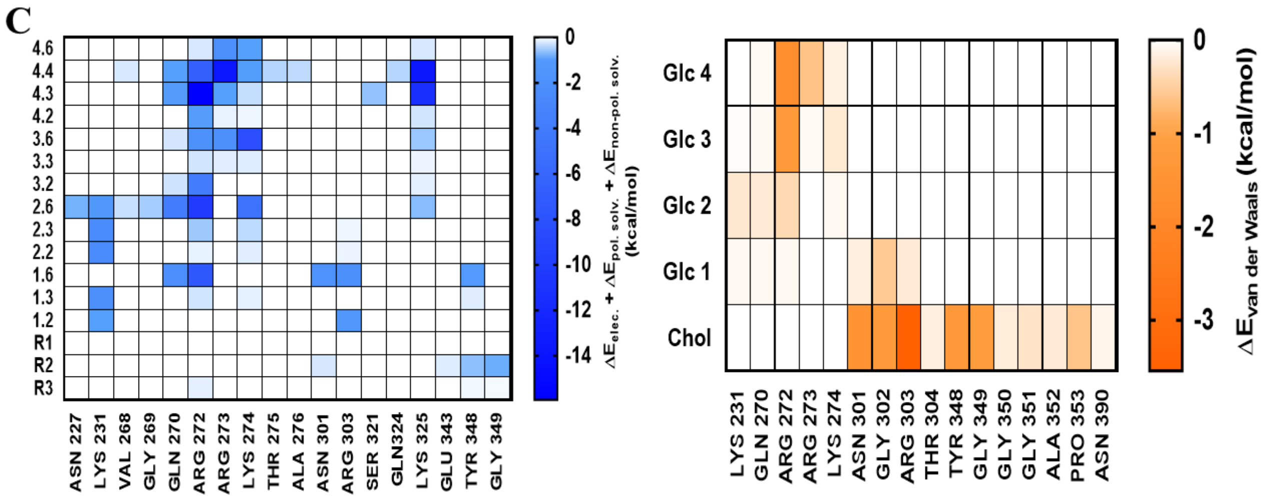

Fig. S9. Calculated running averages of root mean square deviation (RMSD) for each MD simulation of PG545 (conformations a and b) with heparanase.

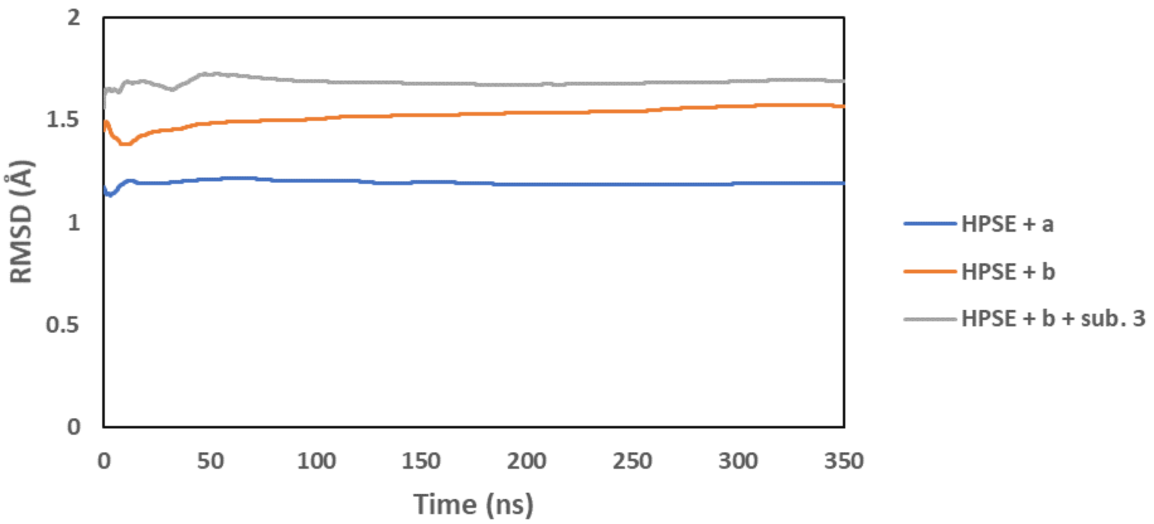

Figure S10. Hydrophobic surface (purple indicates hydrophilic and white tan shows hydrophobic surface) with fondaparinux (**4**) and substrate **3** (in green) in the catalytic pocket where pentasaccharide **4** covers both HBD-1 and HBD-2 binding regions whereas the smaller substrate **3** occupies only the HBD-1 binding region. Hydrophobic surface was rendered using UCSF Chimera version 1.14.

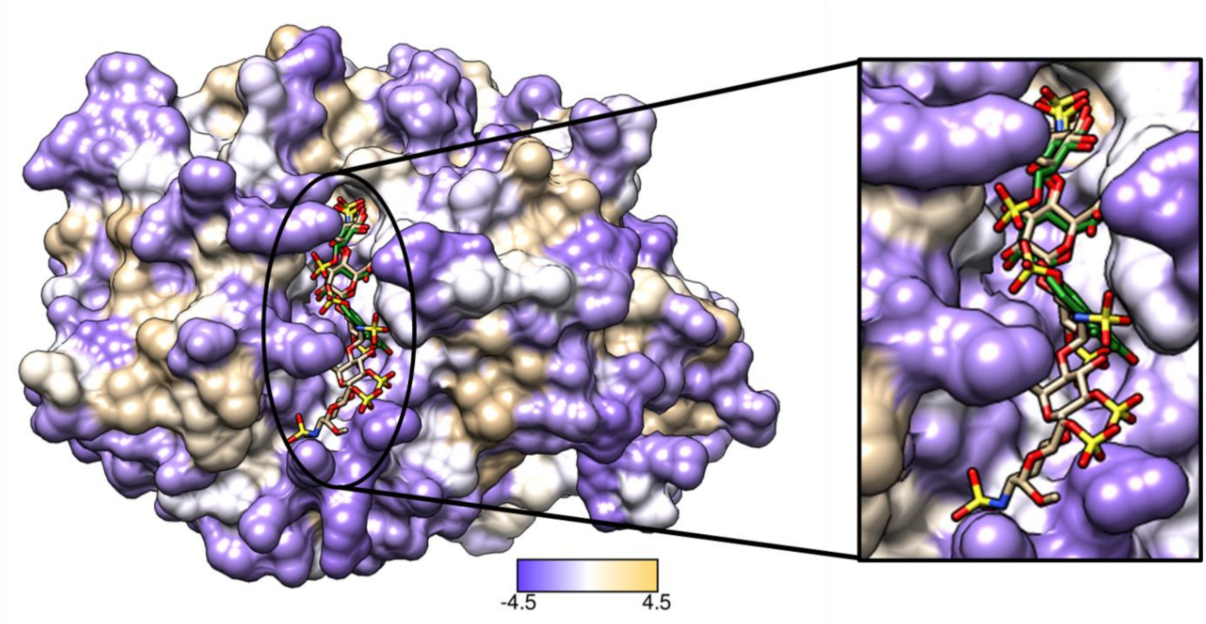

Supplement: Supplementary file 1 — Supporting Information [file CHEM-28-0-s001.pdf]
